# Environmental and life-style risk factors for esophageal squamous cell carcinoma in Africa: a systematic review and meta-analysis

**DOI:** 10.1186/s12889-023-16629-0

**Published:** 2023-09-14

**Authors:** Hannah Simba, Helena Kuivaniemi, Christian C. Abnet, Gerard Tromp, Vikash Sewram

**Affiliations:** 1 Environment and Lifestyle Epidemiology Branch, International Agency for Research on Cancer (IARC/WHO), Lyon, France; 2 African Cancer Institute, Department of Global Health, Faculty of Medicine and Health Sciences, Stellenbosch University, Cape Town, South Africa; 3 Division of Molecular Biology and Human Genetics, Department of Biomedical Sciences, Faculty of Medicine and Health Sciences, Stellenbosch University, Cape Town, South Africa; 4 Division of Cancer Epidemiology and Genetics, National Cancer Institute, Bethesda, USA; 5 DSI-NRF Centre of Excellence for Biomedical Tuberculosis Research, Stellenbosch University, Cape Town, South Africa; 6 Centre for Bioinformatics and Computational Biology, Stellenbosch University, Stellenbosch, South Africa

**Keywords:** Esophageal cancer, Systematic review, Attributable risk, African esophageal cancer corridor, Risk factors

## Abstract

**Background:**

The African Esophageal Squamous Cell Carcinoma (ESCC) corridor, which spans from Ethiopia down to South Africa, is an esophageal cancer hotspot. Disproportionately high incidence and mortality rates of esophageal cancer have been reported from this region. The aim of this study was to systematically assess the evidence on environmental and life-style risk factors associated with ESCC in African populations.

**Methods:**

We followed the Preferred Reporting Items for Systematic Reviews and Meta-Analyses (PRISMA) guidelines and carried out a comprehensive search of all African published studies up to March 2023 using PubMed, Embase, Scopus, and African Index Medicus databases.

**Results:**

We identified 45 studies with measures of association [odds ratio (OR), relative risk (RR), and 95% confidence intervals (95%CI)], which reported on several environmental and lifestyle risk factors for ESCC in Africa. We performed a meta-analysis on 38 studies investigating tobacco, alcohol use, combined tobacco and alcohol use, polycyclic aromatic hydrocarbon exposure, hot food and beverages consumption (which served as a proxy for esophageal injury through exposure to high temperature), and poor oral health. We found significant associations between all the risk factors and ESCC development. Analysis of fruit and vegetable consumption showed a protective effect. Using population attributable fraction (PAF) analysis, we calculated the proportion of ESCC attributable to tobacco (18%), alcohol use (12%), combined tobacco and alcohol use (18%), polycyclic aromatic hydrocarbon exposure (12%), hot food and beverages intake (16%), poor oral health (37%), and fruit and vegetable consumption (-12%).

**Conclusions:**

Tobacco smoking and alcohol consumption were the most studied risk factors overall. Areas where there is an emerging body of evidence include hot food and beverages and oral health. Concurrently, new avenues of research are also emerging in PAH exposure, and diet as risk factors. Our results point to a multifactorial etiology of ESCC in African populations with further evidence on prevention potential.

**Supplementary Information:**

The online version contains supplementary material available at 10.1186/s12889-023-16629-0.

## Background

Esophageal cancer (EC) is a lethal malignancy ranking as the 6^th^ most common cause of cancer mortality worldwide [1]. In 2020, 604,100 new cases and 544,076 deaths were estimated to have occurred, indicative of the high fatality associated with an EC diagnosis [1]. About 80% of EC cases and deaths occur in economically developing countries, where esophageal squamous cell carcinoma (ESCC) is the major subtype contributing approximately 90% of all ECs [2, 3], in contrast to esophageal adenocarcinoma, which is more prevalent in the Western countries [2]. The high mortality rate is attributable to late diagnosis with patients presenting at advanced stage due to a lack of early symptoms.

ESCC has a peculiar geographical distribution with high incidence rates reported from China to Iran, parts of South America and from Eastern to Southern Africa [4, 5]. The variability in incidence between high and low risk areas across the globe has been reported to be up to tenfold [6]. Variations within regions and countries have also been noted. This peculiar distribution draws questions on the specificity of certain risk factors to distinct geographical regions.

The African ESCC corridor, which spans from Kenya down to South Africa on the easterly side of Africa, is an ESCC hotspot region. This African corridor includes Burundi, Malawi, Kenya, Uganda, Tanzania, Zimbabwe, Madagascar, and South Africa [3, 7]. High incidence rates from this corridor have been reported as far back as 1969 [8]. ESCC cases from the African cancer corridor are also reported to be younger than those found elsewhere in the world [9]. These findings suggest that there are possible unique risk factors in this region [3].

EC has a multi-factorial etiology. The risk factors reported worldwide comprise lifestyle, environmental and genetic factors. The lifestyle and environmental factors include smoking, alcohol consumption, poor diet, micronutrient deficiency, exposure to polycyclic aromatic hydrocarbons (PAHs) through cooking and heating methods, esophageal thermal injury from consuming hot foods and beverages, obesity, infectious agents, low socioeconomic status (SES), and exposure to contaminants which have carcinogenic effects [6, 10, 11]. Genetic basis and susceptibility to EC has also been studied, with reports of single nucleotide polymorphisms (SNPs), genomic alterations and epigenetic modifications contributing to tumour development [6, 12, 13]. Familial syndromes, including tylosis and Fanconi anemia, have been reported to be associated with an increased risk of malignancy [6].

Despite advances in the management and treatment, ESCC prognosis is still poor with a survival rate of < 5% in economically developing countries [4]. The etiology of ESCC and the reasons for the high EC burden in Africa are not well understood. The rapid fatality of the cancer, poor prognosis, and contribution of reported modifiable risk factors make ESCC research important in Africa and worldwide. A number of studies in Africa have investigated the association between risk factors and ESCC, with an increase in the number of studies within the last decade. This body of evidence, when systematically assessed and analysed, will shed light on the epidemiology of ESCC in the African populations. It will also substantiate the role of reported risk factors on esophageal carcinogenesis, shed light on emerging risk factors, and provide knowledge on the pathobiology of EC. Improved understanding of EC is required to design better prevention and treatment modalities.

The aim of this systematic review was to provide an in-depth analysis of key environmental and lifestyle factors associated with ESCC development in African populations, and perform a meta-analysis and population attributable fraction (PAF) analysis. Since the last systematic review on ESCC [10], new studies have been published, and further evidence has come to the fore on previous controversial factors where there has not been sufficient power to show an effect. The study aimed to complement the previous systematic reviews [10, 11] by incorporating an updated methodology which included an expanded search strategy, encompassing additional databases and recent publications, and by providing a more up-to-date synthesis of the topic. Furthermore, we included a broader range of analytical methods aiming to address potential gaps in the evidence. Genetic factors were not included in the current study since they were reported recently in a separate study [13]. The aim of this study was achieved through: 1) critical appraisal of African literature on known and emerging risk factors; 2) data synthesis through pooled analysis of each risk factor using meta-analysis; and 3) quantifying contribution of risk factors to disease burden using the PAF analysis.

## Methods

### Study design

The study assessed all environmental and lifestyle risk factors reported in relevant peer-reviewed African literature (cross-sectional, case–control, and cohort studies) and tested for an association with ESCC development or progression. We followed the Preferred Reporting Items for Systematic Reviews and Meta-Analyses (PRISMA) guidelines [14]. To assess the quality of methods and reporting of the published studies, the Joanna Briggs Institute Meta Analysis of Statistics Assessment and Review Instrument (JBI-MAStARI) for Cohort and Case–Control Studies was used [15].

### Data sources, search strategy and extraction

We carried out a literature search on all published African ESCC studies up to March 2023. We developed a comprehensive set of search terms subjectively and iteratively to capture all EC studies carried out in African populations. The search and screening strategy was based on the following framework: population—African individuals, exposure- any factors studied for an association with EC, comparison: N/A, outcome—esophageal cancer, study design—observational studies (cohort, case–control, cross-sectional). Our search did not include the term "risk factor(s)" or any or the individual risk factors named in the manuscript. The goal was to capture all African literature (excluding gray literature) on ESCC and then manually screen the studies to find those which assessed risk factors. Our rationale for doing it this way was to avoid missing any studies that investigated the role of previously unknown factors in ESCC. We excluded animal- and lab-based studies during the screening process. We searched the following electronic bibliographic databases without time or language limits: Medline (PubMed), Embase (OViD), Scopus, African index medicus, and Africa-wide information (EbsCOHost). We also checked the reference lists of potentially relevant articles for additional citations and used the "related citations" search key in PubMed to identify similar papers.

We checked Medline (PubMed) to identify controlled vocabulary (MeSH) terms related to EC, and identified text keywords based on our knowledge of the field [13]. Medline search terms were modified for other electronic databases to conform to their search functions. Search histories are provided in Additional file 1 for all databases used for this study.

**Fig. 1:**
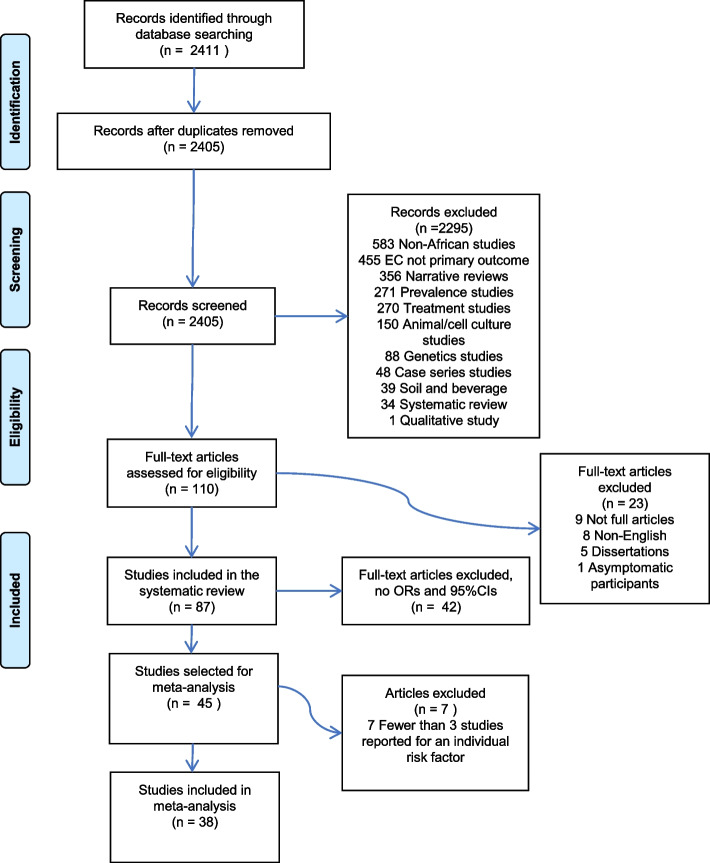
Outline of the study using the PRISMA diagram

Two authors (HS and VS) carried out the screening for eligible studies. First, the two authors read the titles and abstracts independently and then met to finalise an initial list. Full articles of the studies selected based on the initial screening, were read and assessed for inclusion to the systematic review. Figure 1 shows the outline for the selection of eligible studies.

Data extraction was carried out by two authors (HS and VS) using data extraction forms in Microsoft Excel software.

### Assessment of the quality of methods and reporting, and data extraction

The quality of the methods and reporting used in the published studies was assessed using a quality assessment tool adapted from the JBI-MAStARI (Additional file 2) [15]. The assessment included stage of EC in patients, confounding factors, assessment of outcomes in cases and controls, reliability of assessment of outcomes methods, and statistical analysis used. External validity and representativeness of sample to the population was confirmed if the study had at least 150 cases and/or controls. Statistical analysis was assessed by determining if the correct statistical test was used as well as if enough information was reported regarding the analysis methods used. Studies were classified as low quality (score of 1–3), moderate quality (score of 4–6) or high quality (score of 7 +). Only studies, which reported on measures of association [odds ratio (OR), relative risk (RR), and 95% confidence intervals (95%CI)], were assessed for quality of methods and reporting.

### Data analysis

Where data were available from a minimum of three studies, pooled statistical analysis was carried out using meta-analysis and PAF analysis. Where statistical pooling was not possible, the results were presented in a narrative form.

Meta-analysis was performed using the R statistical software [16]. The metagen R package was the main package used for the analysis. A random effects model was used in the analysis, using the Sidik-Jonkman estimator. A test for heterogeneity and between study variance was done as part of the meta-analysis using the chi-squared test. An outlier detection method was used to remove studies with extreme effect sizes from the meta-analysis [17], shown in the results section. If a study’s CI did not overlap with the CI of the pooled effect from the initial meta-analysis, it was considered an outlier. An influence analysis was further performed to detect studies in the meta-analysis which exerted high influence on the overall results. This was done by repeatedly recalculating the results of the meta-analysis, and each time leaving out one study [17]. This allows for better assessment of studies that influence or distort the overall pooled effect. To further explore the robustness of the meta-analyses, Graphic Display of Heterogeneity (GOSH) analysis was done to identify the patterns of effect sizes and heterogeneity in the data [18]. This is a more vigorous and computationally intensive method. A second meta-analysis was done after the removal of outliers. Sensitivity analysis was done using the "Leave-One-Out" influence analysis on studies included in the final meta-analysis. This was done to determine which study may have had an excessive influence on the overall effect size. For the meta-analyses, we selected a single estimate per study and risk factor, representing the effective estimate from the study. Where this was not feasible, and for instances of separate estimates (e.g., men and women, multi-country), we aggregated these estimates into a single meta estimate.

Publication bias analysis was done through funnel plots to determine and visualize whether small studies with small effect sizes are missing from the meta-analysis, and this was visualised through funnel plots. The Egger’s test of the intercept was performed to test for a funnel plot asymmetry.

Attributable risk is used to determine how much of an outcome is attributable to a particular risk factor, and hence provides with estimates (proportions or percentages) of how an outcome can be influenced with the removal or reduction of that risk factor. The PAF was computed using the formula [19]:

$$\sum \frac{p*(RR-1)}{p*(RR-1)+1}$$

where p is the proportion of people in the population exposed to the risk factor, and RR is the relative risk. Where only ORs were reported, we converted OR to RR using data provided in the studies [20]. The formula was executed using a function that we generated in the R statistical software. The overall PAF value for each risk factor was computed using weighted PAF values. The final PAF values where therefore calculated using the following equation, incorporating weighted PAF values:

$$\sum \frac{p}{\sum p}*pAF$$

where p is the proportion of people in the population exposed to the risk factor and ∑p is the sum of p.

We calculated the combined PAF from exposure to tobacco smoking, alcohol consumption, hot food and beverages consumption, PAH, and oral health using the following equation [21]:

$$pAF=1-\left(1-{pAF}_{1}\right)*\left(1-{pAF}_{2}\right)*\left(1-{pAF}_{3}\right)*\left(1-{pAF}_{4}\right)*\left(1-{pAF}_{5}\right)$$

where PAF_1_ is the PAF for tobacco smoking, PAF_2_ is the PAF for alcohol consumption, PAF_3_ is the PAF for hot food and beverages consumption, PAF_4_ is the PAF for PAH exposure, and PAF_5_ is the PAF for oral health. This equation assumes independence of exposure from the five sources.

## Results

### Outline of the systematic review

The initial search produced 2,411 articles, which were screened for duplicates and six duplicates were removed (Fig. 1). The remaining 2,405 articles were screened using titles and abstracts for eligibility. A total of 2,295 articles were removed after the screening, since they were not original observational studies reporting associations between any factors and EC in Africa, with the exception of genetic risk factors. This exclusion was deliberate as we have previously published work on ESCC and genetic risk factors in Africa [13]. Full text assessment was done on the remaining 110 articles. Twenty-three articles were removed for the following reasons: nine had no full text, eight were non-English articles, five were dissertations and one included asymptomatic participants only. Finally, 87 studies we included in the study for appraisal and analysis.

Risk factors reported in the 87 included studies were smoking and alcohol consumption, SES, diet, PAH exposure, consumption of hot food and beverages, oral health, geophagia, infectious agents, esophageal inflammation, family history of cancer and non-acid gastro-esophageal reflux. The studies were published between 1972 and 2023. The diagnostic methods for ESCC used included histopathology, barium swallow, and brush cytology. Of the 87 studies, only 45 (52%) reported association of a risk factor to ESCC using ORs or RRs and 95%CIs.

Quality of methods and reporting assessment was done on the 45 articles that reported ORs or RRs and 95%CIs and hence qualified for quantitative assessment (Additional file 2). The majority of the articles (73%) were of moderate quality (score of 4–6). Six studies (13%) were of low quality (score of 1–3). Six studies (13%) had high quality reporting (score of 7–9). The least reported characteristics were the EC stage of the patients, the response rates of participants and screening for control participants.

### Meta-analysis

Studies that did not report on ORs or RR and 95%CIs were not included in the meta-analysis or the PAF analysis. Also, if fewer than three studies were available for any given risk factor, the risk factor was not assessed in the meta-analysis or PAF analysis, leaving a total of 38 studies on seven different risk factors for these analyses (Fig. 1). Slight differences exist in some of the ORs and CIs presented in the meta-analysis compared to those reported in the original publications due to the random effects model that we used, which can yield different estimates for the ORs and standard errors if the study reported results from a fixed-effects model. However, these differences are small and did not affect the overall trend.

**Fig. 2:**
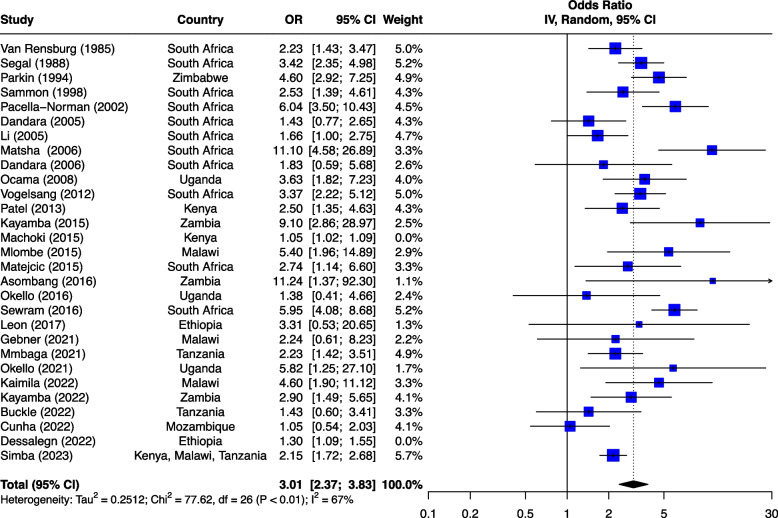
Effect of tobacco use on esophageal cancer in Africa. The forest plot was generated using the R software. Study column gives the first author and the year of the publication. First all studies listed here in chronological order were included in the meta-analysis (Additional file 3). Then studies that were considered outliers were removed from the final meta-analysis by setting their weight to 0%. OR, odds ratio; CI, confidence interval

**Fig. 3:**
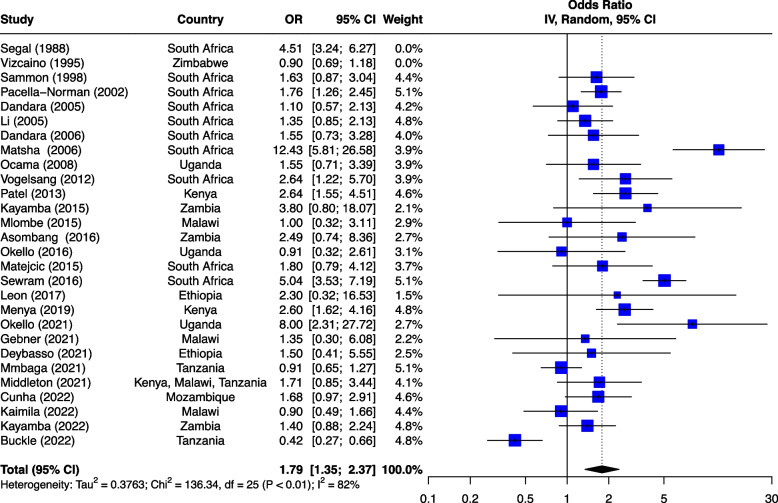
Effect of alcohol consumption on ESCC in Africa. The forest plot was generated using the R software. Study column gives the first author and the year of the publication. Additional file 4 shows the initial analyses before outliers (indicated here with weight of 0%) were removed. OR, odds ratio; CI, confidence interval

**Fig. 4:**
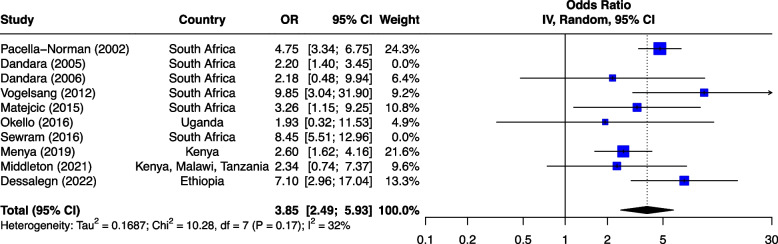
Effect of combined tobacco and alcohol use on ESCC in Africa. The forest plot was generated using the R software. Study column gives the first author and the year of the publication. Additional file 5 shows the initial analyses before outliers (indicated here with weight of 0%) were removed. OR, odds ratio; CI, confidence interval

**Fig. 5:**
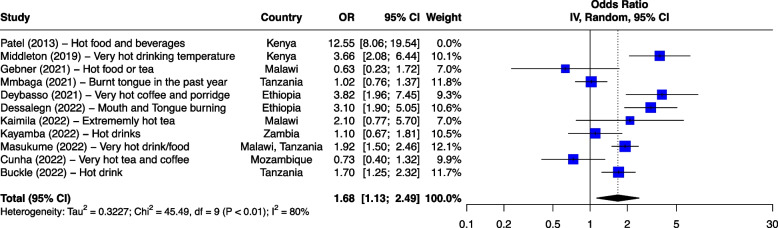
Effect of hot food and beverages on ESCC in Africa. The forest plot was generated using the R software. Study column gives the first author and the year of the publication. Additional file 6 shows the initial analyses before outliers (indicated here with weight of 0%) were removed. OR, odds ratio; CI, confidence interval

**Fig. 6:**
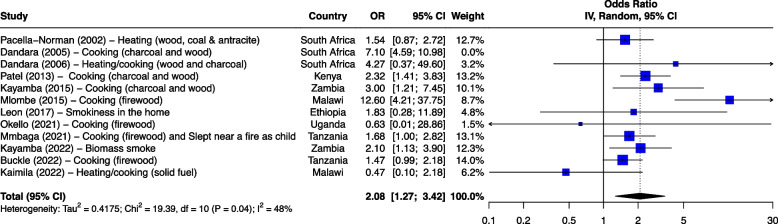
Effect of PAH on ESCC in Africa. The forest plot was generated using the R software. Study column gives the first author and the year of the publication. Additional file 7 shows the initial analyses before outliers (indicated here with weight of 0%) were removed. OR, odds ratio; CI, confidence interval

**Fig. 7:**
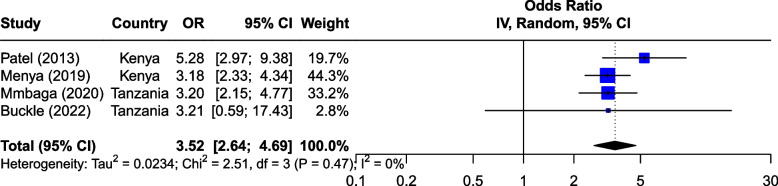
Effect of oral health on ESCC in Africa. The forest plot was generated using the R software. Study column gives the first author and the year of the publication. OR, odds ratio; CI, confidence interval; DMFT, sum of the number of Decayed, Missing due to caries, and Filled Teeth in the permanent teeth; TFI, Thylstrup-Fejerskov index

**Fig. 8:**
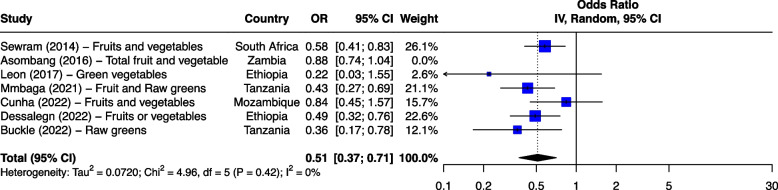
Effect of fruits and vegetables consumption on ESCC in Africa. The forest plot was generated using the R software. Study column gives the first author and the year of the publication. Additional file 8 shows the initial analyses before outliers (indicated here with weight of 0%) were removed. OR, odds ratio; CI, confidence interval

The seven risk factors included in the meta-analysis were: tobacco smoking, alcohol consumption, combined tobacco and alcohol use, consumption of hot food and beverages, fruit and vegetable consumption, oral health and PAH exposure (Table 1). We first analyzed all studies together and then using outlier detection methods, removed the studies with extreme effect sizes from the final meta-analysis. The outliers are still displayed in the meta-analysis forest plots (Figs. 2, 3, 4, 5, 6, 7 and 8), however their weight was set to 0%, indicating that we did not include them in the pooled analysis. Influence analysis was also done to detect studies which were distorting the overall effect size the most as well as to corroborate the results from the outlier detection methods. Three studies [22–24], reported their effect sizes as RR, therefore ORs were calculated from the exposed vs non-exposed data provided in the respective publications and used in our meta-analysis. Forest plots from the initial meta-analysis, without removal of outliers are presented in Additional files 3, 4, 5, 6, 7 and 8 (for oral health only one analysis was performed because the heterogeneity was 0% in the meta-analysis). Baujat plots from outlier, influence and cluster analysis are presented in Additional files 9, 10, 11, 12, 13, 14 and 15. Due to the very large size of the GOSH files, they are not included here and are only available directly from the corresponding author. Sensitivity analysis on the final meta-analysis using the "Leave-One-Out" influence analysis did not show significant deviations from the overall effect size (Additional files 16– 22).

**Table 1:** Studies (*n* = 38) included in the meta-analysis of seven different risk factors

First author (year)	Reference number	Country	Cases (N)	Controls (N)
**Tobacco Use (29 studies)**			**8,425**	**14,329**
Van Rensburg (1985)	[22]	South Africa	211	211
Segal (1988)	[23]	South Africa	200	391
Sammon (1998)	[25]	South Africa	130	130
Parkin (1994)	[26]	Zimbabwe	826	3007
Pacella-Norman (2002)	[27]	South Africa	267	804
Dandara (2005)	[28]	South Africa	244	272
Li (2005)	[29]	South Africa	189	198
Matsha (2006)	[30]	South Africa	92	490
Dandara (2006)	[31]	South Africa	100	94
Ocama (2008)	[32]	Uganda	55	232
Vogelsang (2012)	[33]	South Africa	345	344
Patel (2013)	[34]	Kenya	159	159
Mlombe (2015)	[35]	Malawi	96	180
Kayamba (2015)	[36]	Zambia	50	49
Machoki (2015)	[37]	Kenya	83	166
Matejcic (2015)	[38]	South Africa	463	480
Sewram (2016)	[39]	South Africa	334	621
Okello (2016)	[40]	Uganda	67	142
Asombang (2016)	[41]	Zambia	27	45
Leon (2017)	[42]	Ethiopia	73	133
Gebner (2021)	[43]	Malawi	157	70
Mmbaga (2021)	[44]	Tanzania	471	471
Okello (2021)	[45]	Uganda	31	54
Kayamba (2022)	[46]	Zambia	131	235
Buckle (2022)	[47]	Tanzania	100	108
Kaimila (2022)	[48]	Malawi	300	300
Cunha (2022)	[49]	Mozambique	143	212
Dessalegn (2022)	[50]	Ethiopia	338	338
Simba (2023)	[51]	Kenya, Malawi, Tanzania	830	844
**Alcohol consumption (28 studies)**			**8,516**	**12,041**
Segal (1988)	[23]	South Africa	200	391
Sammon (1998)	[25]	South Africa	130	130
Vizcaino (1995)	[52]	Zimbabwe	881	760
Pacella-Norman (2002)	[27]	South Africa	267	804
Dandara (2005)	[28]	South Africa	244	272
Li (2005)	[29]	South Africa	189	198
Dandara (2006)	[31]	South Africa	145	194
Matsha (2006)	[30]	South Africa	142	105
Ocama (2008)	[32]	Uganda	55	232
Vogelsang (2012)	[33]	South Africa	345	344
Patel (2013)	[34]	Kenya	159	159
Kayamba (2015)	[36]	Zambia	50	49
Matejcic (2015)	[38]	South Africa	463	480
Mlombe (2015)	[35]	Malawi	96	180
Sewram (2016)	[39]	South Africa	334	621
Okello (2016)	[40]	Uganda	67	142
Menya (2019)	[53]	Kenya	422	414
Leon et al. (2017)	[42]	Ethiopia	73	133
Asombang (2016)	[41]	Zambia	27	45
Okello (2021)	[45]	Uganda	31	54
Mmbaga (2021)	[44]	Tanzania	471	471
Middleton (2021)	[54]	Kenya, Malawi, Tanzania	539	539
Deybasso (2021)	[55]	Ethiopia	104	208
Gebner (2021)	[43]	Malawi	157	70
Kayamba (2022)	[46]	Zambia	131	235
Buckle (2022)	[47]	Tanzania	100	108
Kaimila (2022)	[48]	Malawi	300	300
Cunha (2022)	[49]	Mozambique	143	212
**Combined tobacco and alcohol use (10 studies)**			**4,952**	**7,486**
Pacella-Norman (2002)	[27]	South Africa	267	804
Dandara (2005)	[28]	South Africa	244	272
Dandara (2006)	[31]	South Africa	145	194
Vogelsang (2012)	[33]	South Africa	345	344
Matejcic (2015)	[38]	South Africa	463	480
Okello (2016)	[40]	Uganda	67	142
Menya (2019)	[53]	Kenya	422	414
Sewram (2016)	[39]	South Africa	670	1188
Middleton (2021)	[54]	Malawi	539	539
Dessalegn (2022)	[50]	Ethiopia	338	338
**Hot food and beverages consumption (11 studies)**			**3,553**	**3,810**
Patel (2013)	[34]	Kenya	159	159
Middleton (2019)	[56]	Kenya	430	440
Mmbaga (2021)	[44]	Tanzania	471	471
Deybasso (2021)	[55]	Ethiopia	104	208
Gebner (2021)	[43]	Malawi	157	70
Kayamba (2022)	[46]	Zambia	131	235
Buckle (2022)	[47]	Tanzania	100	108
Kaimila (2022)	[48]	Malawi	300	300
Masukume (2022)	[57]	Malawi, Tanzania	310	313
Cunha (2022)	[49]	Mozambique	143	212
Dessalegn (2022)	[50]	Ethiopia	338	338
**PAH exposure (12 studies)**			**2,676**	**4,875**
Pacella-Norman (2002)	[27]	South Africa	267	804
Dandara (2005)	[28]	South Africa	244	272
Dandara (2006)	[31]	South Africa	145	194
Patel (2013)	[34]	Kenya	159	159
Kayamba (2015)	[36]	Zambia	50	48
Mlombe (2015)	[35]	Malawi	96	180
Leon (2017)	[42]	Ethiopia	73	133
Okello (2021)	[45]	Uganda	31	54
Mmbaga (2021)	[44]	Tanzania	471	471
Kayamba (2022)	[46]	Zambia	131	235
Buckle (2022)	[47]	Tanzania	100	108
Kaimila (2022)	[48]	Malawi	300	300
**Fruit and vegetable consumption (7 studies)**			**1,767**	**2,183**
Asombang (2016)	[41]	Zambia	27	45
Leon (2017)	[42]	Ethiopia	73	133
Sewram (2014)	[58]	South Africa	344	621
Mmbaga (2021)	[44]	Tanzania	471	471
Buckle (2022)	[47]	Tanzania	371	363
Cunha (2022)	[49]	Mozambique	143	212
Dessalegn (2022)	[50]	Ethiopia	338	338
**Poor oral health (4 studies)**			**1,227**	**1,228**
Patel (2013)	[34]	Kenya	159	159
Menya (2019)	[59]	Kenya	287	285
Mmbaga (2020)	[60]	Tanzania	310	313
Buckle (2022)	[47]	Tanzania	100	108

#### Tobacco use

Tobacco use was the most commonly investigated risk factor, with 29 (64%) of the 45 included studies reporting quantifiable associations between smoking and EC (Fig. 2). The 29 studies were case–control studies and included 8,425 cases and 14,329 controls. The majority (*n* = 11) of the studies were from South Africa, and the rest were from Ethiopia (*n* = 2), Malawi (*n* = 3), Tanzania (*n* = 2), Zambia (*n* = 3), Zimbabwe (*n* = 1), Kenya (*n* = 2), Uganda (*n* = 3), Mozambique (*n* = 1), and one study reporting on a multicenter case–control study from Malawi, Kenya and Tanzania. The studies were published between 1985 and 2023. Most studies indicated an increased risk of ESCC in people who use tobacco, with ORs ranging from 1.05 to 11.24 [37, 41]. The highest risk was reported in studies done on two Zambian populations and a South African female population, with ORs of 11.24 (1.37–92.30 95%CI), 9.10 (2.86–28.97), and 11.10 (4.50–27.00 95%CI), respectively [30, 36, 41]. The rest of the studies stated increased risk of ESCC with ORs ≤ 6.27. In studies that assessed the tobacco as a risk factor separately for men and women, men had a slightly higher risk than women [27, 39, 51]. The effect of snuff use was also investigated in one Kenyan study [34], two South African studies [25, 27], a Zimbabwean study [26] and one multi-site case–control study from Malawi, Kenya and Tanzania [51]. Overall, the effect was weaker than tobacco smoking in all studies.

The pooled analysis for tobacco use showed an effect size of OR of 3.01 (2.37–3.83 95%CI) (Fig. 2). Heterogeneity (I^2^) of 67% with *p* < 0.01 was recorded after removal of two studies. Egger’s test did not reveal any funnel plot asymmetry (*p* = 0.38). One of the studies included in this analysis [37] is not indexed on PubMed®.

#### Alcohol consumption

Alcohol consumption was investigated as a risk factor in 28 of the 45 (62%) studies with quantifiable associations to ESCC (Fig. 3). All the studies were case–control studies with the majority (*n* = 10) of the studies performed in South Africa, and three each from Uganda, Zambia, and Malawi, two each from Ethiopia and Kenya, one each from Mozambique, and Zimbabwe, whilst one study reported on a multicenter case–control study in Malawi, Kenya and Tanzania. All studies combined included 8,516 cases and 12,041 controls. There was a significant overlap with the studies reporting the effects of tobacco smoking. The studies were published between 1988 and 2023.

The highest risk was reported in a study done on a South African population with OR of 15.40 (5.48–43.26 95%CI) for men and 9.90 (3.41–28.71 95%CI) for women consuming commercial beer [30]. Another South African study reported an OR of 5.09 (3.42–7.58 95%CI) and 3.89 (2.49–6.08 95%CI) for traditional beer and commercial spirits, respectively [23]. One of the studies by Patel et al. [34], after adjusting for sex, age, smoking, snuff use, and cooking and sleeping in the same room, alcohol consumers were 45% more likely to have ESCC compared to those who did not consume alcohol. Three studies from Malawi did not show a significant association between alcohol use and ESCC [35, 48, 54].

The pooled analysis for alcohol consumption demonstrated an effect size of OR 1.79 (1.35–2.37 95%CI) (Fig. 3), indicating that alcohol users are almost twice as likely to develop ESCC compared to non-alcohol users. The test for heterogeneity showed I^2^ of 82% (*p* < 0.01) after removal of two studies. Egger’s test did not reveal any funnel plot asymmetry (*p* = 0.60).

#### Tobacco and alcohol

A combination of smoking and alcohol as a risk factor for ESCC was investigated in 10 of the 45 studies (Fig. 4). They included 4,952 cases and 7,486 controls. The combination of tobacco and alcohol was reported to increase the risk in most studies with ORs ranging from 1.95 to 19.06 [28, 30, 33, 38, 54]. A South African study described an increased risk of OR 18.20 (8.10–41.70 95%CI) for women, which was significantly higher than OR of 3.50 (1.50–8.40 95%CI) for men [30]. In another study which assessed the risk for men and women separately, the risk for women was slightly higher, with an OR of 4.80 (3.00–7.80 95%CI), than for men, 4.70 (2.80–7.90 95%CI) [27]. One study from Malawi [54] did not show a significant association.

The pooled analysis of combined alcohol and tobacco had an effect size of OR 3.85 (2.49–5.93 95%CI) (Fig. 4), indicating that individuals who use both alcohol and tobacco users are approximately four times more likely to develop ESCC compared to non-alcohol and tobacco users. Test for heterogeneity showed I^2^ of 32% (*p* = 0.17) after removal of two studies. Egger’s test did not show the presence of funnel plot asymmetry (*p* = 0.74).

#### Hot food and beverages

Consumption of hot food and beverages serves as a proxy for esophageal injury through exposure to high temperature and it is thus plausible to combine the consumption of hot food and the consumption of hot beverages into one entity providing an indirect measure of thermal injury to the esophagus. This risk factor was reported in eleven case–control studies (3,553 cases and 3,810 controls) (Fig. 5). These included two studies from Malawi, two from Ethiopia, two from Tanzania, two from Kenya, one from Mozambique, one from Zambia, and one multisite case control study from Malawi and Tanzania. The highest ORs were reported from a Kenyan study where drinking hot beverages and eating hot food increased the risk of ESCC with OR of 12.78 (6.95–23.50 95%CI) and 12.30 (6.46–23.44 95%CI), respectively [34]. The rest of the studies (*n* = 7) with statistically significant associations had ORs ranging from 1.40 (drinking hot tea in Kenya) [56] to 5.10 (drinking very hot coffee in Ethiopia) [55]. Three additional studies from Kenya, Ethiopia, Tanzania and Malawi have also reported that the consumption of hot tea, hot food and hot chai are important risk factors for ESCC [7, 61, 62]. However, these studies did not report risk estimates. A case control study from Ethiopia reported that drinking water during meals had a protective effect on ESCC development [63].

An overall effect size estimate of OR 1.68 (1.13–2.49 95%CI) and I^2^ of 80% (*p* < 0.01) was obtained for the pooled analysis of hot food and beverages exposure, showing that consumption of hot food and beverages doubles the risk of developing ESCC (Fig. 5). One study was removed from the analysis. Egger’s test did not indicate the presence of funnel plot asymmetry (*p* = 0.86).

#### PAH exposure

Twelve case control studies reported PAH exposure as a risk factor for ESCC (Fig. 6). They included 2,676 cases and 4,875 controls. Indoor air pollution was assessed through smokiness in the home, heating and cooking fuel, sleeping near a fire, and mursik (a fermented milk beverage which may contain charcoal), and were classified as PAH exposures. Three studies were from South Africa,  two from Tanzania, two from Zambia, two from Malawi, one from Ethiopia, one from Kenya and one from Uganda. Ten studies reported significant associations between PAH exposure and ESCC with OR ranging from 1.54 to 15.20. In a South African study, the use of wood and charcoal for heating and cooking was reported to increase ESCC risk with OR 15.20 (8.17–28.27 95%CI) [31]. A Kenyan study reported on the use of mursik, the consumption of which increased the risk of ESCC with OR 3.72 (1.95–7.10 95%CI) [34]. Sleeping near a fire as a child showed a borderline significance in a Tanzanian study with OR of 1.28 (0.94–1.75 95%CI) [44].

In the pooled analysis, a forest plot of PAH exposure showed an effect estimate of OR of 2.08 (1.27–3.42 95%CI) (Fig. 6). Test for heterogeneity showed an I^2^ of 48% (*p* = 0.04) after removal of one study. Egger’s test gave *p* = 0.57 and did not indicate the presence of funnel plot asymmetry.

#### Oral health

Two Kenyan and two Tanzanian case–control studies explored oral health as a risk factor for ESCC (Fig. 7). They included 1,227 cases and 1,228 controls. A study by Patel et al. [34] showed that tooth loss was associated with an increased risk of ESCC with OR 5.28 (2.97–9.38 95%CI). Tooth loss was also associated with an increased risk of ESCC in a study by Menya et al. [59] in Kenya and by Mmbaga et al. [60] in Tanzania. In the Kenyan study, other components of oral health were assessed which showed an increased risk, these include: decayed teeth (≥ 3) OR 4.40 (3.22–6.02 95%CI), brushing teeth only once per week OR 2.30 (0.98–5.39 95%CI), never having brushed teeth OR 2.50 (1.02–6.12 95%CI), oral leukoplakia OR 3.10 (1.81–5.32 95%CI), and the sum of number of decayed + missing + filled teeth ≥ 8 OR 3.00 (1.49–6.05 95%CI) [59]. The Tanzanian study reported similar components and results followed a similar direction. This study also reported that the use of charcoal to clean teeth increased the risk of ESCC with OR 2.33 (1.33–4.08 95%CI) [60]. Less frequent than daily teeth cleaning was associated with increased ESCC risk [47].

Pooled analysis of the association between poor oral health and ESCC development showed an overall estimate of OR 3.52 (2.64–4.69 95%CI). An I^2^ of 0% (*p* < 0.47) was recorded without removing any studies. Egger’s test did not indicate the presence of funnel plot asymmetry.

#### Diet

Fifteen studies investigated the effect of diet on ESCC. This included food groups, food items, beverages, vitamins and trace elements. A total of 1,767 cases and 2,183 controls were assessed for fruit and vegetable consumption. This included seven case–control studies from Ethiopia (*n* = 2), South Africa (*n* = 1), Mozambique (*n* = 1), Tanzania (*n* = 2), and Zambia (*n* = 1).

Consumption of fruits, vegetables and green legumes was individually associated with a protective effect to ESCC development in all studies (Fig. 8). In a South African study by Sewram et al. [58], eating fruits 5–7 times a week was associated with a protective effect of OR 0.51 and 0.42 for men and women, respectively. Consumption of vegetables 5–7 times a week, also had a protective effect of OR 0.62 and 0.50 for men and women, respectively. One Ethiopian study [42] reported that not eating vegetables at least once a week, or not eating green vegetables at all significantly increased the risk of ESCC with OR of 12.68 (1.99–80.96 95%CI) and 400 (12.00–13,345 95%CI), respectively [42]. Another study from Ethiopia reported that eating fruits or vegetables daily reduced the risk of ESCC with OR of 0.49 [50]. Studies done in Mozambique [49], Tanzania [44, 47], and Zambia [41] also showed a protective effect of fruit and vegetable consumption to ESCC development.

Three South African studies reported increased risk of ESCC in participants who consume wild vegetables [24, 25, 58]. The wild vegetables comprised imifino, Uthyuthu (*Amaranthus thunbergii)*, imbikicane (*Chenopodium album*), and umsobo (*Sofanum nigrum*). One of the studies on South African women reported an increased risk of ESCC in consumers of wild imifino vegetables with OR of 1.84 (1.04–3.27 95%CI) [58]. The highest risk was observed by Sammon et al. [24] with a RR of 2.86 (1.16–8.00 95%CI).

Other food items that were reported to increase the risk of ESCC development, were: purchased maize, pumpkin, beans, sorghum and porridge reported in three South African and one Ethiopian study [22, 25, 58, 64]. One Ethiopian study [42], also indicated that saltiness in food increased the risk of ESCC with OR of 7.79 (1.21–50.30 95%CI). In another South African study, daily and weekly consumption of margarine was reported to have a protective effect with OR of 0.51 and 0.71, respectively [22]. One Ethiopian study in increased risk of ESCC associated with consumption of a homemade non-alcoholic drink called kennetoo, which is reported to contain acrylamide due to the way it is made [55]. Schaafsma et al. [65] performed an ecological study assessing the ESCC development and six micronutrients (calcium, copper, iodine, magnesium, selenium, zinc) in 32 African countries. Iron, zinc and selenium were described to have a protective effect in males and females, whilst magnesium was reported to be protective in females only.

Pooled analysis for fruit and vegetables consumption resulted in an overall OR of 0.51 (0.37–0.71 95%CI) and I^2^ of 0% (*p* = 0.42) (Fig. 8). One study was removed from the analysis. Egger’s test indicated the presence of funnel plot asymmetry with *p* = 0.45.

### Systematic review for risk factors without pooled analysis

Below we summarize the results on the seven other ESCC risk factors for which we identified published studies, but pooled analysis was not possible due to the small number of studies for each of them. We also excluded some risk factors from pooled analysis if they would not have provided informative or meaningful results due to differences in the measurement or assessment of the risk factors. The risk factors without pooled analyses were: esophageal injury, non-acid gastroesophageal reflux, SES, infectious agents, water source, family history of cancer, and geophagia.

#### Esophageal inflammation and injury

Esophageal inflammation and injury due to self-induced vomiting and caustic ingestion was reported as a risk factor in two South African studies and one Kenyan study. The case–control studies had a total of 661 cases and 266 controls. These studies were excluded from pooled analysis due to the differences in exposure assessments. In the South African study, induced vomiting was associated with ESCC, reporting OR of 1.83 (1.13–2.96 95%CI) [66]. The study reported on various methods used by the participants to induce vomiting which included the use of salt water, traditional medicine, warm water, holy water, and vinegar water. The South African case–control study did not show a statistically significant association between induced vomiting or use of traditional emetics and ESCC development [24]. The Kenyan study reported that caustic ingestion was associated with an increased risk of ESCC with OR 11.35 (3.04–42.46 95%CI) [37]. The use of traditional medicines, which can be used as emetics, was investigated in a South African case–control study by Sammon et al. [24], but no association between traditional medicines and EC development was found.

#### Non-acid gastroesophageal reflux

Non-acid gastroesophageal reflux was reported to increase the risk of ESCC in a South African case–control study with OR of 8.80 (3.20–24.50 95%CI) [67]. The authors measured non-acid gastroesophageal reflux using a digi-trapper high-definition multichannel impedance and medical measurement system for pH, which involved placing a test catheter near the esophagogastric junction for 24 h. Sample size was very small with only 32 cases and 49 controls. Non-acid gastroesophageal reflux was reported in 23 (73%) of the cases and in 11 (22%) of the controls.

#### Socio-economic status

Low SES was assessed as a risk factor for ESCC development in eleven case–control studies. One study was from South Africa, one from Malawi, two from Tanzania, one from Uganda, one from Zambia, one from Mozambique, one from Zimbabwe, one from Ethiopia, and two were from Kenya. Pooled analysis was not performed because the method used to measure SES varied across studies. Additionally some measures were not considered suitable for capturing the multidimensional, cultural and contextual nature of SES. Overall low SES was associated with increased risk of ESCC. SES was measured using salaries/household income [23, 34, 47, 49], occupational status [52, 64], assets owned [45, 46], international wealth index score [44, 47], SES score [48], and type of housing [37, 46]. The South African study reported an increased risk of ESCC associated with lower salaries, and found RR ranging from 1.23 to 74.94 for various low-salary levels [23]. In the Zimbabwean study low occupational status and mining as an occupation were found to increase the risk for ESCC when compared to high occupational status in men with OR of 1.50 and 2.50, respectively [52]. One Kenyan study showed that a monthly salary of over 100 dollars reduced the risk of ESCC with OR of 0.59 (0.46–0.77 95%CI) [34]. The second Kenyan study showed that poor housing increased the risk of ESCC with OR of 1.98 (1.11–3.53 95%CI) [37].

#### Infectious agents

Human papillomavirus (HPV) and human immunodeficiency virus (HIV) infection were assessed as risk factors in six studies (two Zambian, two Malawian, one Tanzanian, and one South African). Pooled analysis was not performed due to the significant diversity among the infectious agents studied, which made it inappropriate to group them into a single category for a pooled analysis. HPV was assessed in two studies, a South African study [68] which reported a statistically significant association with ESCC with OR 1.59 (1.19–2.13 95%CI), and a Zambian study which showed no association [36]. The Zambian study [36] also assessed the association between HIV infection and ESCC and found a significant association (OR 2.30, 1.00–5.10 95%CI), however, another Zambian study found no association between HIV infection and ESCC [41]. Similarly in Malawi, one study [48] reported an association between HIV infection and ESCC with OR of 4.20 (1.90–9.40 95%CI), whilst another study [43] showed no association. A Tanzanian study showed no association between HIV status and ESCC [44]. Other infectious agents reported in the Malawian study [43] included oral thrush, *Mycobacterium tuberculosis*, *Herpes zoster*, *Helicobacter pylori*, *Herpes simplex*, cytomegalovirus, Epstein–Barr virus, and *Varicella zoster*, none of which showed an association with ESCC development.

#### Water source

Water source was assessed as a risk factor for ESCC development in case–control studies of a total of 1,032 cases and 1,146 controls from Kenya, Tanzania, and Zambia [44, 46, 59]. The use of spring/river water compared to piped/rain water was reported to be associated with ESCC development in Kenya with OR 3.10 (1.50–6.50 95%CI) [59]. The Tanzanian and Zambian studies showed no significant associations between ESCC and water source. Pooled analysis was not performed due to differences in the selection of a reference category in the studies.

#### Family history of cancer

Family history of cancer was analysed in five case–control studies with 1,329 cases and 1,508 controls. It was reported to increase the risk of ESCC in study participants in Kenya [37] (OR 3.50, 1.29–9.49 95%CI), in Tanzania [44] (OR 2.30, 1.04–5.08 95%CI), in Tanzania [47] with participants over 45 years old (OR 4.03, 1.36–11.98), and in Malawi [48] (OR 2.50, 1.00–5.90 95%CI). Pooled analysis was not performed due to the variation in degree of relatedness and difference cancer types in relatives.

#### Geophagia

Geophagia was reported in three case–control studies and one multicenter case–control study (1,333 cases and 1,402 controls). A Tanzanian study [44] investigated consumption of soil as a child and ESCC risk and reported a significant association with OR 1.67 (1.09–2.55 95%CI). A study from Malawi [48] reported statistically significant association between geophagia and ESCC (OR 1.80, 1.20–2.80 95%CI). A Zambian study reported no association between geophagia and ESCC. In a multicenter case control study (Tanzania, Malawi, Kenya) and the most comprehensive study on ESCC and geophagia thus far, non-significant increase in ESCC risk (OR 1.66; 0.77–3.55 95%CI) was reported in Tanzanian women who consumed soil during pregnancy and regularly [69]. Results from Malawi and Tanzania did not show a significant association between women who consumed soil during pregnancy or regularly, and ESCC development. Pooled analysis was not performed because of variations in exposure measurement.

### Population attributable fraction (PAF)

PAF calculations were done for seven risk factors: tobacco smoking, alcohol consumption, combined tobacco and alcohol use, hot food and beverage consumption, oral health, fruit and vegetable consumption, and PAH exposure. The data were taken from the 38 studies selected for the meta-analysis. Seven studies were excluded from the analysis due to not having enough information on exposure. The PAF attributable to tobacco smoking was 18%, whilst for alcohol consumption it was 12%. According to our analysis, the combined exposure of tobacco and alcohol attributed 18% of the ECs. Consumption of hot food and beverages was responsible for 16% of ESCC cases. Exposure to PAHs contributed 12% of ESCC cases. Poor oral health attributed 37% of ESCC cases in our results. Fruit and vegetable consumption, due to its protective effect, showed a negative PAF of –12%. Our estimates show that 66% of ESCC cases are attributable to the combined effects of tobacco smoking, alcohol consumption, esophageal injury and PAH exposure.

## Discussion

EC constitutes a major health burden in specific geographic regions of the African esophageal cancer corridor. We performed a systematic review that identified studies which reported tobacco use, alcohol consumption, SES, diet, PAH exposure, consumption of hot food and beverages, oral health, infectious agents, esophageal injury, family history of cancer, water source, geophagia, and non-acid gastro-esophageal reflux as environmental and life-style risk factors for ESCC in Africa using risk estimates. Our results point to a multifactorial etiology of ESCC which was also reported in two other previous systematic reviews [10, 11]. Most of the studies in our systematic review were reported from the African Esophageal Cancer Corridor. A meta-analysis of 38 independent studies was done for the following seven risk factors, individually, which had a sufficient number of studies for a pooled analysis: tobacco smoking, alcohol consumption, combined smoking and alcohol use, PAH exposure, hot food and beverages, oral health as well as fruit and vegetable consumption. Our study also aggregated data from additional sources other than PubMed and performed a PAF analysis, which has not been done before. The meta-analysis provided information on the overall risk of a specific risk factors to ESCC, whilst the PAF is the proportional reduction in disease that would occur in a population if the exposure to a risk factor were modified or removed.

### Systematic review and meta-analysis

Data from western countries have conclusively implicated tobacco smoking and alcohol consumption as the main risk factors for ESCC [6]. In our study, tobacco smoking was the most studied risk factor and emerged as a plausible contributing agent for ESCC with OR of 3.01 (2.37–3.83 95%CI). In a systematic review on the effect of tobacco smoking and ESCC, Asian studies showed a slightly smaller OR of 2.31 (1.78–2.99 95% CI), whilst European studies showed higher OR of 4.21 (3.13–5.66 95%CI) [70]. Alcohol consumption was the second most reported risk factor and our meta-analysis had OR 1.79 (1.35–2.37 95%CI). Our results showed a weaker association compared to results from a meta-analysis on Asian (OR 5.05, 3.40–7.49 95%CI) and European studies (OR 3.42, 2.29–5.09 95%CI) [70]. Several studies from Malawi, Tanzania, Uganda and Ethiopia showed a borderline or no significant association between ESCC and alcohol use. Strong associations were evident between home-brewed beer and ESCC. The preparation of this beer is often done in oil drums which may contain iron and other carcinogens [71].

Some previous studies reported the interaction between tobacco and alcohol, with high risk estimates, indicative of synergistic effects of combined exposure to tobacco and alcohol. However, because none of these studies were originally designed as interaction studies, they may not have had enough statistical power to detect interactions. In a systematic review and meta-analyses by Prabhu et al. [72] pooled analysis of five studies from Asian populations showed a positive synergistic effect of tobacco and alcohol exposure. The synergy factor was reported as an OR of 3.28 (2.11–5.08 95%CI), Cochrane’s Q P value = 0.05 and I^2^ = 55.3% [72]. The authors reported that the combined effect of tobacco and alcohol exposure was approximately twice that of each risk factor alone [72]. In our study, pooled analysis of combined tobacco and alcohol use showed similar findings with OR 3.85 (2.49–5.93 95%CI) and an I^2^ of 32%. The results support the synergistic effects of combined alcohol and tobacco exposure reported in literature, with a two-fold increase in risk compared to alcohol consumption alone. Regarding tobacco smoking alone, the combined effect of tobacco smoking and alcohol consumption increases ESCC risk by one factor.

Poor oral health (i.e., teeth loss, dental fluorosis, teeth decay, and low frequency of teeth brushing) emerged as an important risk factor for ESCC in our meta-analysis, with OR of 3.52 (2.64–4.69 95%CI). Our results corroborated a meta-analysis from Asia which reported that teeth loss was associated with an increased risk of EC (OR 1.52) and high frequency of teeth brushing was associated with a lower incidence of EC (OR 0.62) [73]. In a Chinese case–control study (616 patients and 770 controls) in 2017, tooth loss and low frequency of teeth brushing increased ESCC risk [74]. Changes in the oral microbiota, associated with poor oral health, have been suggested as the underlying mechanism [74].

Over the past few years, there has been an increase in the number of African studies assessing the role of hot food and beverages in the ESCC pathogenesis. It serves as a proxy for esophageal injury through exposure to high temperature and it is thus plausible to combine the consumption of hot food and the consumption of hot beverages into one entity providing an indirect measure of thermal injury to the esophagus. These studies reported an association, corroborated with other studies in China, Iran and South America which demonstrated the same effect [11]. Additionally, results from our meta-analysis were consistent with these findings, showing that the consumption of hot food and beverages was associated with an increased risk of ESCC of almost twofold.

Our study assessed PAHs from different sources (heating and cooking fuel, and consumption of charcoal powder when drinking mursik) and found that PAHs increase ESCC risk. In a systematic review on the role of biomass fuel (wood, charcoal, coal, dung, and crop residues) in ESCC development, the use of biomass fuel for heating and cooking was associated with ESCC development, due to the smoke exposure [75]. The highest risk was reported in Africa and Asia. These results were corroborated in our study, as pooled analysis demonstrated that PAH exposure was associated with increased risk of developing ESCC with OR of 2.08 (1.27–3.42 95%CI). Similar to our study, low SES was reported to be associated with increased ESCC risk in Indian, American and Swedish studies [76–78].

Consumption of fruits and vegetables reduced the risk of ESCC in our study. This evidence is supported by a previous meta-analysis [79] on 32 studies on Asian, European, North and South American populations. The study reported that consumption of vegetables and fruits was associated with significantly reduced risk of ESCC with summary RRs of 0.56 (0.45–0.69 95%CI) and 0.53 (0.44–0.64 95%CI), respectively [79]. Our pooled analysis for fruit and vegetable consumption among African populations showed OR 0.51 (0.37–0.71 95%CI). Fruit and vegetable consumption had a protective effect on ESCC, reducing the risk of developing ESCC by 45%. Due to the small number of studies, fruit and vegetable consumption could not be analysed separately. The role of micronutrient deficiencies in the etiology of ESCC is contested in the literature. A study included in our systematic review, one of the biggest micronutrient studies done in 32 African countries, iron, zinc and selenium were described to have a protective effect in males and females, whilst magnesium was reported to be protective in females only [65]. The study was an ecological study, which is an observational study where data are analyzed for entire populations in different geographical regions at a single point in time, however, ecological studies are relevant mainly for hypothesis generation. In a systematic review on micronutrients and EC [80] increased dietary intake of total iron and zinc, and reduced heme iron intake was reported to be protective against EC. The use of cross sectional case–control studies to examine the role of diet in EC is known to bias estimates away from the null, so the reported associations should be interpreted with caution [81]

### Population attributable fraction

Our study provides evidence-based assessment of the proportion of ESCC cases attributable to certain risk factors. To quantify the contribution of a risk factor to the development of ESCC, PAF analysis was performed. Our analysis showed that tobacco smoking and alcohol consumption contributed to 18% and 12% of the ESCC cases, respectively, whilst their combined exposure contributed 18% of the ESCC cases. In a Chinese study, the contribution of alcohol consumption in EC cases was reported to have a PAF of 10.9% (15.2% for men and 1.3% for women) [82]. Another Chinese study reported the combined contribution of tobacco smoking and alcohol consumption in ESCC as 40.9% [83]. In a meta-analysis of large-scale population-based cohort studies in Japan, higher PAF values of 55.4%, 61.2%, and 81.4% for smoking, alcohol consumption, and combined smoking and alcohol consumption, respectively, were reported [84]. An Australian study in 2013 reported that PAF for ESCC due to tobacco smoking was 49% and for alcohol consumption it was 32% [85]. Attributable fraction for ESCC was reported in a Pakistani population with the following PAF values: chewing areca nut (10.8%), chewing betel quid with tobacco (47.6%), oral snuff (10.1), and cigarette smoking (22.3) [86]. Tobacco chewing and snuff use are common practices in African populations, but understudied.

Poor oral health emerged as an important risk factor, and interestingly, had the highest PAF of 37% among all the risk factors. This finding underscores the importance of more comprehensive studies investigating the role of poor oral health in the African Esophageal Cancer Corridor and the underlying mechanism to pathogenesis. Consumption of hot food and beverages had a PAF of 16%. In a 2003 study done in Paraguay, maté consumption, which is normally consumed at high temperatures, had a population-attributable risk of 53% [87]. However, the authors mention that two competitive mechanisms could explain this high attributable risk: the high temperature of the mate causing thermal injury of the esophagus, and the carcinogenic effects of the herbs used in preparation of the drink. Whilst our study did not assess low intake of fruits and vegetables, this has been shown in an Australian study to attribute to 9% of ESCC cases [85], and 2% in a Canadian study [88].

The combined attributable risk of the five most significant risk factors (tobacco smoking, alcohol consumption, poor oral health, consumption of hot food and beverages and PAH exposure) was estimated to be 66%. Assuming that each of these exposures are mutually exclusive, this suggests that 66% of ESCC cases would be prevented if these exposures were removed. However, it is likely that most of these factors are related and therefore the true combined PAH is likely smaller.

The cumulative estimate is therefore an upper bound based on independence of individual factors. If there is an interaction, such as between smoking and alcohol, some or all of the effect of one of the factors can be subsumed by the other [21] For instance, the PAF for tobacco use is 18%, whilst for alcohol use it is 12% and the combined PAF is 18%. Overall, our results show that certain risk factors are population- and region-specific, and point to a multifactorial etiology of ESCC.

### Strengths and limitations

The strength of this study is that it provides the most comprehensive meta-analysis on environmental risk factors associated with ESCC in the African populations to date. It is also the first study to perform an aggregated PAF analysis of multiple risk factors which contribute to ESCC cases in the African populations and thus able to inform esophageal health policy and cancer control and planning in the region.

One of the main limitations was the heterogeneity of the original studies. Studies assessing the same risk factor often had a different study design, geographical location, exposure measurement, exposure assessment category, exposure intensity, and confounding factors adjusted for, and variability in sample size (most studies were small case–control studies), therefore caution is needed when interpreting the magnitude of the risk estimates. Similar limitations regarding differences in exposure assessment quality were reported in a recent systematic review on the carcinogenicity opium consumption [89] None of the studies directly measured carcinogen levels in tobacco, alcohol or indoor smoke. Acetaldehyde levels of alcohol may have differed, especially in home-brewed beer.

Several studies did not report risk estimates, and hence could not be included in the meta-analyses. Most of these studies were published before 1990. There were some deficiencies in the quality of reporting and methods in some of the studies. About one third of the studies did not report on adjusting for confounding factors. Unmeasured and unadjusted confounders can result in a false association.

There is a lack of interaction studies to investigate the multifactorial etiology of ESCC. There are a number of gene-environment interaction studies which have been published [90] assessing the interaction between genetic variants and family history of cancer, age, sex, food hygiene, eating habits [91], tobacco smoking [92, 93], and alcohol consumption [92–94]. However, the majority of the studies have been published on Asian populations.

Whilst we reported on PAFs, there are potential biases in our estimates due to the uncertainty of the magnitude (RR) of the effect given the sparse literature and the lack of population-based estimates of exposure. Additionally, for risk factors such as alcohol consumption and tobacco smoking, sex-aggregated data would have given a clearer picture of the attributable risk as prevalence of exposure may differ according to sex. Both the meta-analysis and PAF analysis combined fruits and vegetables as one factor, however, the nutritional value of fruits and vegetables differs [95]. The combination of these factors was done due to limited number of studies available for analyses.

While this study employed a comprehensive search strategy, it is important to note that gray literature was not included. This exclusion was a deliberate choice made to uphold methodological rigor and minimize potential bias. Although this decision ensures the reliability of the included studies from reputable, peer-reviewed sources, it may have limited the incorporation of additional perspectives and ongoing research.

We also acknowledge the challenges that come with doing research in low-to-middle-income countries. These include the availability of resources and infrastructure to perform research, including time and expenses. There is also a lack of suitable methods and technologies, which results in the use of non-standardized assessment tools. Most data collection tools are based on self-reporting of lifestyle behaviors and environmental exposures via questionnaires which have a low precision of accuracy and can result in recall and misclassification bias [96]. It is important to highlight the lack of prospective cohort studies, which have the capability of significantly reducing some of the biases common in case–control studies, which is most acutely relevant to studies of diet.

## Conclusions

Overall, our study showed that there is a relatively large body of evidence for smoking and alcohol being associated with ESCC in the African populations, compared to other risk factors. Areas where there is an emerging body of evidence include hot food and beverages, and oral health. Concurrently, new avenues of research are also emerging in PAH exposure, and diet as risk factors. However, studies investigating the etiology of ESCC in Africa are still very limited, therefore more research needs to be done to understand the high prevalence seen in the African Esophageal Cancer Corridor. A standardized way of measuring risk factors will allow for future systematic reviews to report with certainty pooled estimates which can be generalized to the region. The results of our study point to a multifactorial etiology, which includes multiple environmental and life-style risk factors, playing a role in the ESCC risk. Our results have important implication for ESCC control and prevention.

### Supplementary Information

**Additional file 1.** Search histories for systematic review and meta-analysis. List of all queries used for searching PubMed, Embase, Cinahl, Scopus, Web of Science, and African index medicus. PDF.

**Additional file 2.** Quality assessment of included studies. This table summarizes how the quality of reporting and methods was assessed in the studies included in this systematic review and meta-analysis. PDF.

**Additional file 3.** Effect of tobacco use on esophageal cancer in Africa. This diagram is a forest plot showing pooled analysis of all studies (no exclusion of outliers). Study ID gives the first author and the year of the publication. OR, odds ratio; CI, confidence interval. PDF.

**Additional file 4.** Effect of alcohol consumption on ESCC in Africa. This diagram is a forest plot showing pooled analysis of all studies (no exclusion of outliers). Study ID gives the first author and the year of the publication. OR, odds ratio; CI, confidence interval. PDF.

**Additional file 5.** Effect of combined tobacco and alcohol use on ESCC in Africa. This diagram is a forest plot showing pooled analysis of all studies (no exclusion of outliers). Study ID gives the first author and the year of the publication. OR, odds ratio; CI, confidence interval. PDF.

**Additional file 6.** Effect of hot food and beverage on ESCC in Africa. This diagram is a forest plot showing pooled analysis of all studies (no exclusion of outliers). Study ID gives the first author and the year of the publication. OR, odds ratio; CI, confidence interval. PDF.

**Additional file 7.** Effect of PAH on ESCC in Africa. This diagram is a forest plot showing pooled analysis of all studies (no exclusion of outliers). Study ID gives the first author and the year of the publication. OR, odds ratio; CI, confidence interval; PAH, polycyclic aromatic hydrocarbons. PDF.

**Additional file 8.** Effect of fruit and vegetable consumption on ESCC in Africa. This diagram is a forest plot showing pooled analysis of all studies (no exclusion of outliers). Study ID gives the first author and the year of the publication. OR, odds ratio; CI, confidence interval. PDF.

**Additional file 9.** Baujat plot for tobacco use from outlier and influence analysis. PDF.

**Additional file 10.** Baujat plot for alcohol use from outlier and influence analysis. PDF.

**Additional file 11.** Baujat plot for combined tobacco and alcohol use from outlier and influence analysis. PDF.

**Additional file 12.** Baujat plot for hot food and beverage consumption from outlier and influence analysis. PDF.

**Additional file 13.** Baujat plot for PAH exposure from outlier and influence analysis. PAH, polycyclic aromatic hydrocarbons. PDF.

**Additional file 14.** Baujat plot for oral health from outlier and influence analysis. PDF.

**Additional file 15.** Baujat plot for fruit and vegetable consumption from outlier and influence analysis. PDF.

**Additional file 16.** Sensitivity plot for tobacco use. Sensitivity analysis was done using the "Leave-One-Out" influence analysis on studies included in the final meta-analysis. This was done to determine which study may have had an excessive influence on the overall effect size. PDF.

**Additional file 17.** Sensitivity plot for alcohol use. PDF.

**Additional file 18.** Sensitivity plot for combined alcohol and tobacco use. PDF.

**Additional file 19.** Sensitivity plot for hot food and beverage consumption. PDF.

**Additional file 20.** Sensitivity plot for combined PAH exposure. PDF.

**Additional file 21.** Sensitivity plot for oral health. PDF.

**Additional file 22.** Sensitivity plot for fruit and vegetable consumption. PDF.

## Data Availability

The datasets used and/or analyzed during the current study as well as the GOSH plots generated as part of this study are available from the corresponding author.

## Abbreviations

95% CI: 95% Confidence interval
EC: Esophageal cancer
ESCC: Esophageal squamous cell carcinoma
GOSH: Graphic Display of Heterogeneity
HIV: Human immunodeficiency virus
HPV: Human papillomavirus
JBI-MAStARI: Joanna Briggs Institute Meta Analysis of Statistics Assessment and Review Instrument
OR: Odds ratio
PAF: Population attributable fraction
PAH: Polycyclic aromatic hydrocarbons
PRISMA: Preferred Reporting Items for Systematic Reviews and Meta-Analyses
RR: Relative risk
SES: Socioeconomic status
SNP: Single nucleotide polymorphism
