# Supplementary material for: Environmental and life-style risk factors for esophageal squamous cell carcinoma in Africa: a systematic review and meta-analysis
Source: BMC Public Health. 2023 Sep 14;23:1782. doi: 10.1186/s12889-023-16629-0 (PMC10500769; doi:10.1186/s12889-023-16629-0)
Supplement: Supplementary file 1 — Additional file 1. Search histories for systematic review and meta-analysis. List of all queries used for searching PubMed, Embase, Cinahl, Scopus, Web of Science, and African index medicus. PDF. [file 12889_2023_16629_MOESM1_ESM.pdf]

Additional File 1: Search histories for systematic review and meta-analysis	
**Pubmed (MEDLINE)**	
Search	Query
#1	Search cancer or carcinoma or neoplasm*
#2	Search cancer or carcinoma or neoplasm* Field: Title/Abstract
#3	Search Esophageal or oesophageal Field: Title/Abstract
#4	Search esophagus Field: Title/Abstract
#5	Search (#2) AND #3 Field: Title/Abstract
#6	Search (#4) AND #2 Field: Title/Abstract
#7	Search (#5) OR #6 Field: Title/Abstract
#8	Search "Esophageal cancer " Field: Title/Abstract
#9	Search "oesophageal cancer " or “oesophageal neoplasm*” Field: Title/Abstract
#10	Search "Esophageal Neoplasms"[Mesh]
#11	Search "Esophageal Neoplasms" Field: Title/Abstract
#12	Search “Esophageal squamous cell carcinoma*” or “Oesophageal squamous cell carcinoma*” or ESCC Field: Title/Abstract
#13	Search (((((#12) OR #11) OR #10) OR #9) OR #8) OR #7 Field: Title/Abstract
#14	Search "Africa"[Mesh] Field: Title/Abstract
#15	Search algeria OR angola OR benin OR botswana OR burkina faso OR burundi OR cameroon OR cape verde OR central african republic OR chad OR comoros OR congo OR "Democratic Republic of Congo" OR DRC OR djibouti OR equatorial guinea OR egypt OR eritrea OR ethiopia OR gabon OR gambia OR ghana OR guinea OR bissau OR ivory coast OR (Cote d' Ivoire) OR jamahiriya OR kenya OR lesotho OR liberia OR Libya OR madagascar OR malawi OR mali OR mauritania OR mauritius OR mayotte OR morocco OR mozambique OR namibia OR niger OR nigeria OR principe OR reunion OR rwanda OR "Sao Tome" OR senegal OR seychelles OR "Sierra Leone" OR somalia OR "South Africa" OR st helena OR sudan OR swaziland OR eswatini OR tanzania OR togo OR tunisia OR uganda OR zaire OR zambia OR zimbabwe OR "Central Africa" OR "West Africa" OR "East Africa" OR "Southern Africa" OR "South Africa" Field: Title/Abstract
#16	Search (#14) OR #15 Field: Title/Abstract
#17	Search (#16) AND #13 Field: Title/Abstract
**Embase**	
S1	(cancer or carcinoma or neoplasm*).ab. or (cancer or carcinoma or neoplasm*).ti.
S2	(Esophageal or oesophageal or esophagus or oesophagus).ab. or (Esophageal or oesophageal or esophagus or oesophagus).ti.
S3	1 and 2
S4	"Esophageal cancer ".ab. or "Esophageal cancer ".ti.
S5	("oesophageal cancer" or "oesophageal neoplasms").ab. or ("oesophageal cancer" or "oesophageal neoplasms").ti.

S6	"Esophageal squamous cell carcinoma".ab. or "Esophageal squamous cell carcinoma".ti.
S7	("oesophageal squamous cell carcinoma" or ESCC).ab. or ("oesophageal squamous cell carcinoma" or ESCC).ti.
S8	esophagus cancer/
S9	3 or 4 or 5 or 6 or 7 or 8
S10	exp Africa/ or africa.mp.
S11	exp "Africa south of the Sahara"/
S12	10 or 11
S13	(algeria or angola or benin or botswana or " burkina faso" or burundi or cameroon or "cape verde" or "central african republic" or chad or comoros or congo).mp. [mp=title, abstract, heading word, drug trade name, original title, device manufacturer, drug manufacturer, device trade name, keyword, floating subheading word, candidate term word]
S14	("Democratic Republic of Congo" or DRC or djibouti or "equatorial guinea" or egypt or eritrea or ethiopia or gabon or gambia or ghana or guinea or bissau or "ivory coast" or "Côte d' Ivoire").mp.
S15	(jamahiriya or kenya or lesotho or liberia or Libya or madagascar or malawi or mali or mauritania or mauritius or mayotte or morocco or mozambique or namibia or niger or nigeria or principe or reunion or rwanda).mp.
S16	("Sao Tome" or senegal or seychelles or "Sierra Leone" or somalia or "South Africa" or st helena or sudan or swaziland or tanzania or togo or tunisia or uganda or zaire or zambia or zimbabwe).mp.
S17	("Central Africa" or "West Africa" or "East Africa" or "Southern Africa").mp.
S18	12 or 13 or 14 or 15 or 16 or 17
S19	9 and 18
**Cinahl (EBSCOHost)**	
S11	S6 AND S10
S10	S7 OR S8 OR S9
S9	TI (algeria OR angola OR benin OR botswana OR burkina faso OR burundi OR cameroon OR cape verde OR central african republic OR chad OR comoros OR congo OR "Democratic Republic of Congo" OR DRC OR djibouti OR equatorial guinea OR egypt OR eritrea OR ethiopia OR gabon OR gambia OR ghana OR guinea OR bissau OR ivory coast OR (Côte d' Ivoire) OR jamahiriya OR kenya OR lesotho OR liberia OR Libya OR madagascar OR malawi OR mali OR mauritania OR mauritius OR mayotte OR morocco OR mozambique OR namibia OR niger OR nigeria OR principe OR reunion OR rwanda OR "Sao Tome" OR senegal OR seychelles OR "Sierra Leone" OR somalia OR "South Africa" OR st helena OR sudan OR swaziland OR tanzania OR togo OR tunisia OR uganda OR zaire OR zambia OR zimbabwe OR "Central Africa" OR "West Africa" OR "East Africa" OR "Southern Africa" OR "South Africa"

S8	MW africa
S7	AB africa OR TI africa
S6	S1 OR S2 OR S3 OR S4 OR S5
S5	MW esophageal cancer
S4	TI ("oesophageal squamous cell carcinoma" or ESCC) OR AB ("oesophageal squamous cell carcinoma" or ESCC)
S3	TI "Esophageal squamous cell carcinoma" OR AB "Esophageal squamous cell carcinoma"
S2	AB (cancer or carcinoma or neoplasm*) AND AB (Esophageal or oesophageal or esophagus or oesophagus)
S1	TI (cancer or carcinoma or neoplasm*) AND TI (Esophageal or oesophageal or esophagus or oesophagus)
**Scopus**	
((TITLE-ABS-KEY ((cancer OR carcinoma OR neoplasm*) AND (esophageal OR oesophageal OR esophagus OR oesophagus))) OR (TITLE-ABS-KEY ("Esophageal squamous cell carcinoma")) OR (TITLE-ABS-KEY ("oesophageal squamous cell carcinoma" OR escc))) AND ((TITLE-ABS-KEY ("Central Africa" OR "West Africa" OR "East Africa" OR "Southern Africa")) OR (TITLE-ABS-KEY ("Sao Tome" OR senegal OR seychelles OR "Sierra Leone" OR somalia OR "South Africa" OR "st helena" OR sudan OR swaziland OR tanzania OR togo OR tunisia OR uganda OR zaire OR zambia OR zimbabwe)) OR (TITLE-ABS-KEY (mayotte OR morocco OR mozambique OR namibia OR niger OR nigeria OR principe OR reunion OR rwanda)) OR (TITLE-ABS-KEY ("Democratic Republic of Congo" OR drc OR djibouti OR "equatorial guinea" OR egypt OR eritrea OR ethiopia)) OR (TITLE-ABS-KEY (" burkina faso" OR burundi OR cameroon OR "cape verde" OR "central african republic" OR chad OR comoros OR congo)) OR (TITLE-ABS-KEY (algeria OR angola OR benin OR botswana)) OR (TITLE-ABS-KEY (ethiopia OR gabon OR gambia OR ghana OR guinea OR bissau OR ivory AND coast OR (côte AND d' AND ivoire) OR jamahiriya OR kenya OR lesotho OR liberia OR libya OR madagascar OR malawi OR mali OR mauritania OR mauritius)) OR (TITLE-ABS-KEY (africa)))	
**Web of Science**	
S11	#6 AND #10
S10	#7 OR #8 OR #9
S9	TI=(algeria OR angola OR benin OR botswana OR burkina faso OR burundi OR cameroon OR cape verde OR central african republic OR chad OR comoros OR congo OR "Democratic Republic of Congo" OR DRC OR djibouti OR equatorial guinea OR egypt OR eritrea OR ethiopia OR gabon OR gambia OR ghana OR guinea OR bissau OR ivory coast OR (Côte d' Ivoire) OR jamahiriya OR kenya OR lesotho OR liberia OR Libya OR madagascar OR malawi OR mali OR mauritania OR mauritius OR mayotte OR morocco OR mozambique OR namibia OR niger OR nigeria OR principe OR reunion OR rwanda OR "Sao Tome" OR senegal OR seychelles OR "Sierra Leone" OR

	somalia OR "South Africa" OR st helena OR sudan OR swaziland OR tanzania OR togo OR tunisia OR uganda OR zaire OR zambia OR zimbabwe OR "Central Africa" OR "West Africa" OR "East Africa" OR "Southern Africa" OR "South Africa")
S8	KP=(africa)
S7	(TI=(africa)) OR AB=(africa)
S6	#1 OR #2 OR #3 OR #4 OR #5
S5	KP=("esophageal cancer")
S4	(TI=("oesophageal squamous cell carcinoma" or ESCC)) OR AB=("oesophageal squamous cell carcinoma" OR ESCC)
S3	(TI=(Esophageal squamous cell carcinoma)) OR AB=(Esophageal squamous cell carcinoma)
S2	(AB=(cancer or carcinoma or neoplasm)) AND AB=(Esophageal or oesophageal or esophagus or oesophagus)
S1	(TI=(cancer or carcinoma or neoplasm)) AND TI=(Esophageal or oesophageal or esophagus or oesophagus)
**African index medicus**	
"esophageal cancer" or “oesophageal cancer”	
(tw:("esophageal carcinoma ")) AND (tw:("oesophageal carcinoma"))	
