# Supplementary material for: Environmental and life-style risk factors for esophageal squamous cell carcinoma in Africa: a systematic review and meta-analysis
Source: BMC Public Health. 2023 Sep 14;23:1782. doi: 10.1186/s12889-023-16629-0 (PMC10500769; doi:10.1186/s12889-023-16629-0)
Supplement: Supplementary file 2 — Additional file 2. Quality assessment of included studies. This table summarizes how the quality of reporting and methods was assessed in the studies included in this systematic review and meta-analysis. PDF. [file 12889_2023_16629_MOESM2_ESM.pdf]

Additional file 1 — Quality Assessment of Included Studies. This table summarizes how the quality of reporting and methods was assessed in the studies included in this systematic review and meta-analysis.

**Study First author & Year (PMID)**	**Is the sample representative of patients in the population as a whole?**	**Are the patients at a similar point in the course of their condition /illness?**	**Has bias been minimised in relation to cases and of controls?**	**Are confounding factors identified and strategies to deal with them stated?**	**Description of ESCC diagnosis**	**Were controls screened and tested for ESCC?**	**Were response rates reported?**	**Were outcomes measured in a reliable way?**	**Was appropriate statistical analysis used?**	**Score (total number of Y)**
Asombang 2016 (26973419)	N	U	Y	U	Y	Y	N	Y	Y	5
Astini et al 1990 (2083189)	N	U	Y	N	Y	N	N	Y	Y	4
Buckle et al 2022 (35113655)	Y	U	Y	Y	Y	N	N	Y	Y	6
Cunha et al 2022 (36200014)	Y	U	N	Y	Y	N	N	Y	Y	5
Dandara et al 2005 (15978331)	Y	U	Y	N	Y	Y	N	Y	Y	6
Dandara et al 2006 (16272171)	Y	U	Y	Y	Y	N	N	Y	Y	6
Dessalegn et al 2022 (36203447)	Y	N	N	Y	Y	Y	N	Y	Y	5
Deybasso et al 2021 (33664594)	Y	U	Y	Y	Y	N	N	Y	Y	6
Geßner et al 2021 (34342770)	N	U	N	U	Y	N	N	N	Y	2
Kaimila et al 2022	Y	U	Y	Y	Y	N	Y	Y	Y	7
Kayamba 2015 (25641622)	N	U	Y	Y	Y	N	N	Y	Y	5
Kayamba et al 2022 (36158985)	Y	U	Y	Y	Y	N	N	Y	Y	6

Leon et al 2017 (28594883)	N	U	Y	Y	Y	N	N	Y	Y	5
Li et al 2005 (15899651)	Y	U	Y	Y	Y	N	N	Y	Y	6
Machoki et al 2015	N	U	Y	N	Y	N	N	Y	Y	4
Masukume et al 2022 (35768549)	Y	U	Y	Y	Y	N	Y	Y	Y	7
Matejcic et al 2015 (26447020)	Y	U	Y	Y	Y	N	N	Y	Y	6
Matsha b et al 2006 (16607430)	Y	N	NA	N	Y	NA	N	Y	N	3
Matsha et al 2006 (17176219)	Y	U	N	Y	Y	N	N	N	Y	4
Menya b et al 2019 (30582155)	Y	U	Y	Y	Y	N	N	Y	Y	6
Menya et al 2019 (30117158)	Y	Y	Y	Y	Y	N	N	Y	Y	7
Middleton et al 2019 (30496610)	Y	N	Y	Y	Y	N	N	Y	Y	6
Middleton et al 2022 (34921758)	Y	U	Y	Y	Y	U	Y	Y	Y	7
Mlombe et al 2015 (26715952)	N	N	Y	Y	Y	N	N	Y	Y	5
Mmbaga et al 2020 (33320959)	Y	U	Y	Y	Y	N	Y	Y	Y	7
Mmbaga et al 2021 (33144280)	Y	U	Y	Y	Y	N	N	Y	Y	6
Narh et al 2021 (34004024)	Y	U	U	Y	Y	N	N	Y	Y	5
Ocama et al 2008 (19357755)	N	U	U	Y	Y	N	N	Y	Y	4
Okello et al 2016 (27400987)	N	U	U	Y	Y	Y	N	Y	Y	5
Okello et al 2021 (35342792)	N	U	Y	Y	Y	Y	N	Y	Y	6
Pacella-Norman 2002 (12087462)	Y	U	U	Y	Y	U	N	Y	Y	5
Parkin et al 1994 (7827583)	Y	U	U	Y	Y	U	N	Y	Y	5

Patel et al 2013 (24490085)	Y	U	Y	Y	N	N	N	Y	Y	5
Sammon 1992 (1735077)	N	U	Y	N	Y	N	N	N	Y	4
Sammon et al 1998 (9690530)	N	U	Y	U	Y	N	N	N	Y	3
Schaafsma et al 2015 (26448405)	Y	N	NA	Y	U	NA	NA	U	Y	3
Segal et al 1988 (3219281)	Y	U	Y	N	N	N	N	U	Y	3
Sewram et al 2014 (24877989)	Y	U	Y	Y	N	N	Y	Y	Y	6
Sewram et al 2016 (26900781)	Y	U	Y	Y	Y	N	Y	Y	Y	7
Shewaye et al 2016	Y	U	N	U	Y	N	N	Y	Y	4
Simba et al 2023 (36733225)	Y	U	Y	Y	Y	N	N	Y	Y	6
Sitas et al 2007 (17331260)	U	N	U	Y	N	U	N	U	Y	2
van Rensburg et al 1985 (3970816)	Y	U	Y	Y	N	N	N	U	Y	4
Vizcaino et al 1995 (7669592)	Y	N	U	Y	Y	U	N	Y	Y	5
Vogelsang et al 2012 (22623965)	Y	U	Y	Y	Y	N	N	Y	Y	6

ESCC, esophageal squamous cell carcinoma; N, no; NA, not applicable; U, unknown; Y, yes
