# Supplementary figures and images for: Environmental and life-style risk factors for esophageal squamous cell carcinoma in Africa: a systematic review and meta-analysis
Source: BMC Public Health. 2023 Sep 14;23:1782. doi: 10.1186/s12889-023-16629-0 (PMC10500769; doi:10.1186/s12889-023-16629-0)

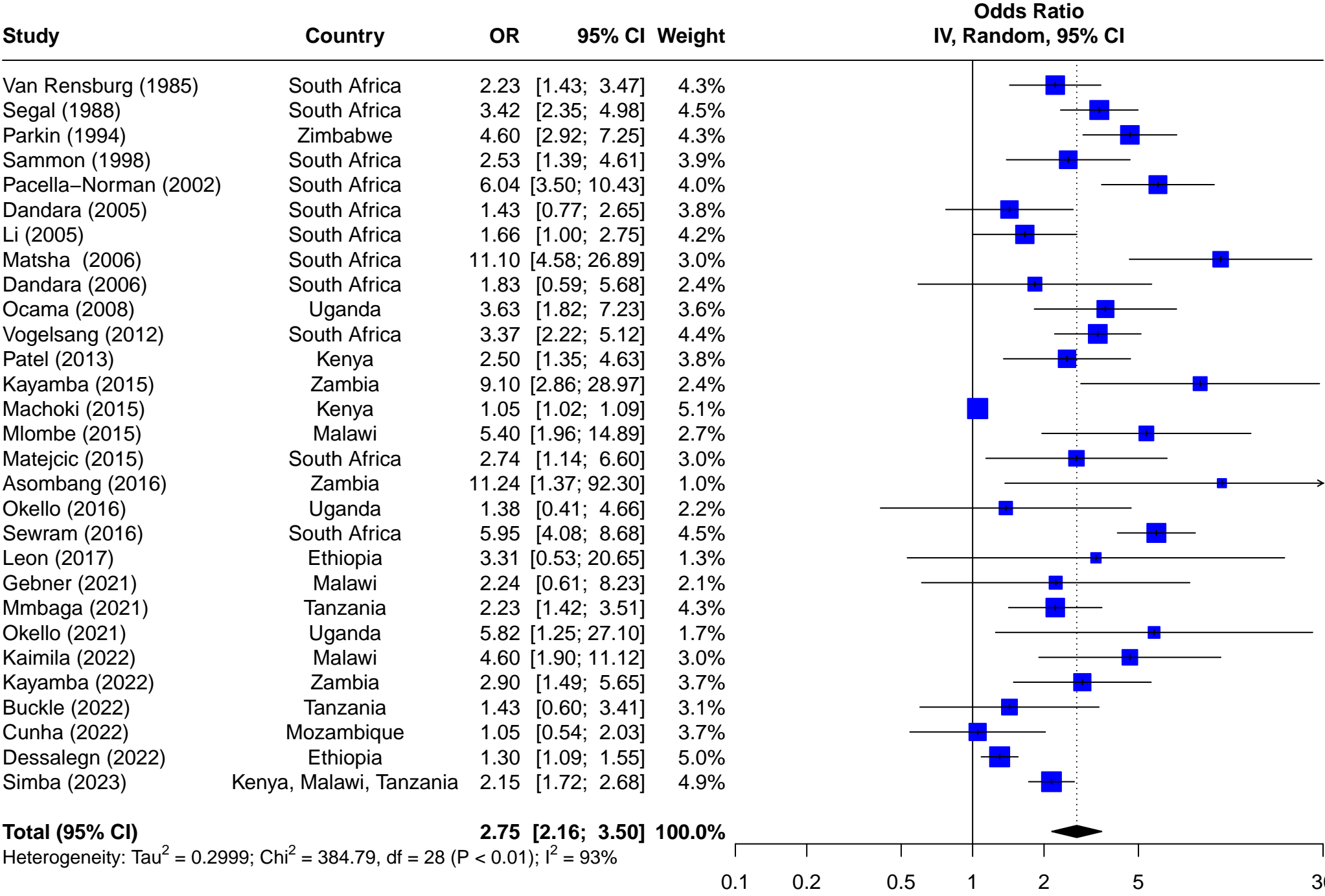

Supplement: Supplementary file 3 — Additional file 3. Effect of tobacco use on esophageal cancer in Africa. This diagram is a forest plot showing pooled analysis of all studies (no exclusion of outliers). Study ID gives the first author and the year of the publication. OR, odds ratio; CI, confidence interval. PDF. [file 12889_2023_16629_MOESM3_ESM.pdf]

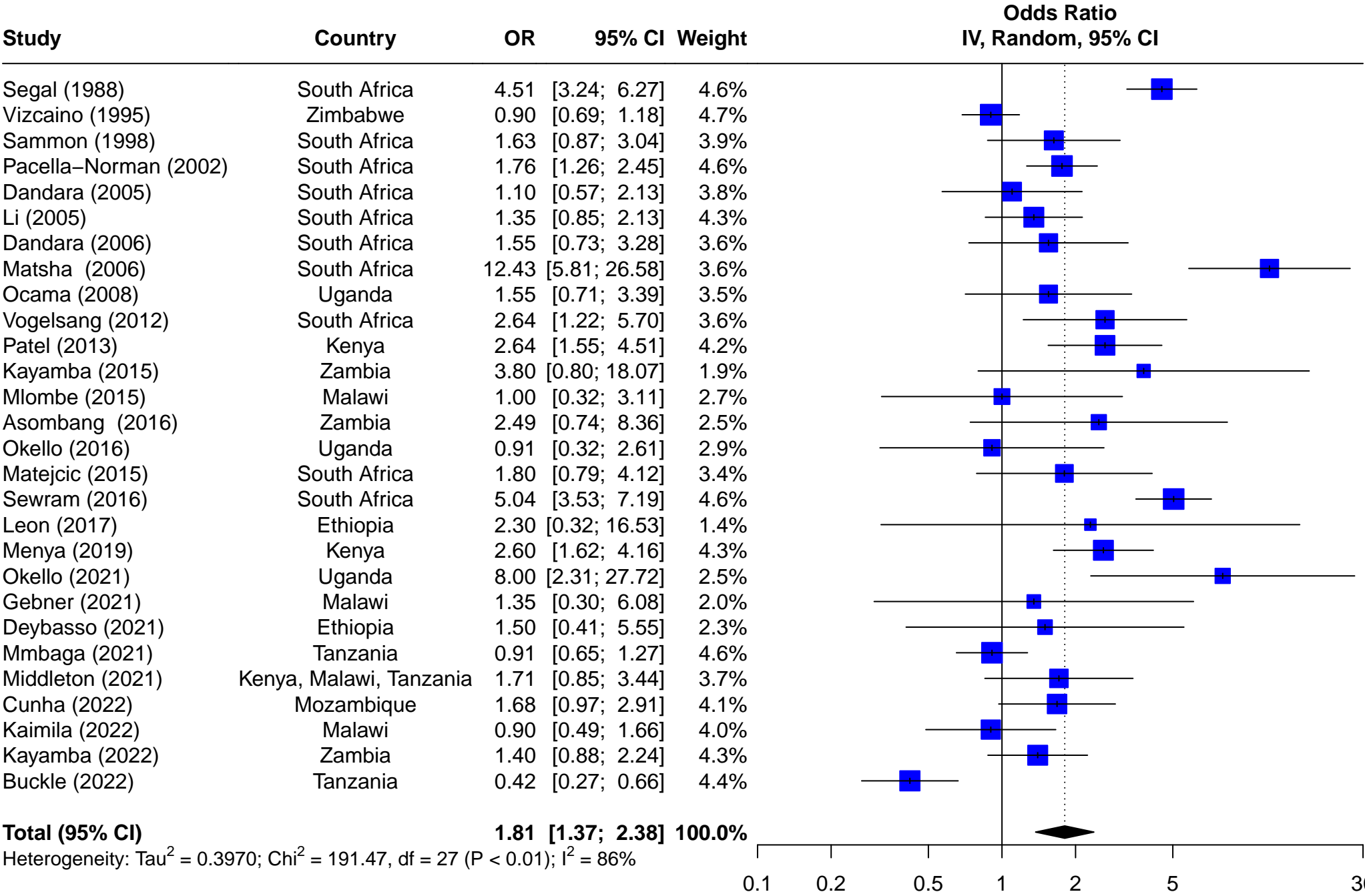

Supplement: Supplementary file 4 — Additional file 4. Effect of alcohol consumption on ESCC in Africa. This diagram is a forest plot showing pooled analysis of all studies (no exclusion of outliers). Study ID gives the first author and the year of the publication. OR, odds ratio; CI, confidence interval. PDF. [file 12889_2023_16629_MOESM4_ESM.pdf]

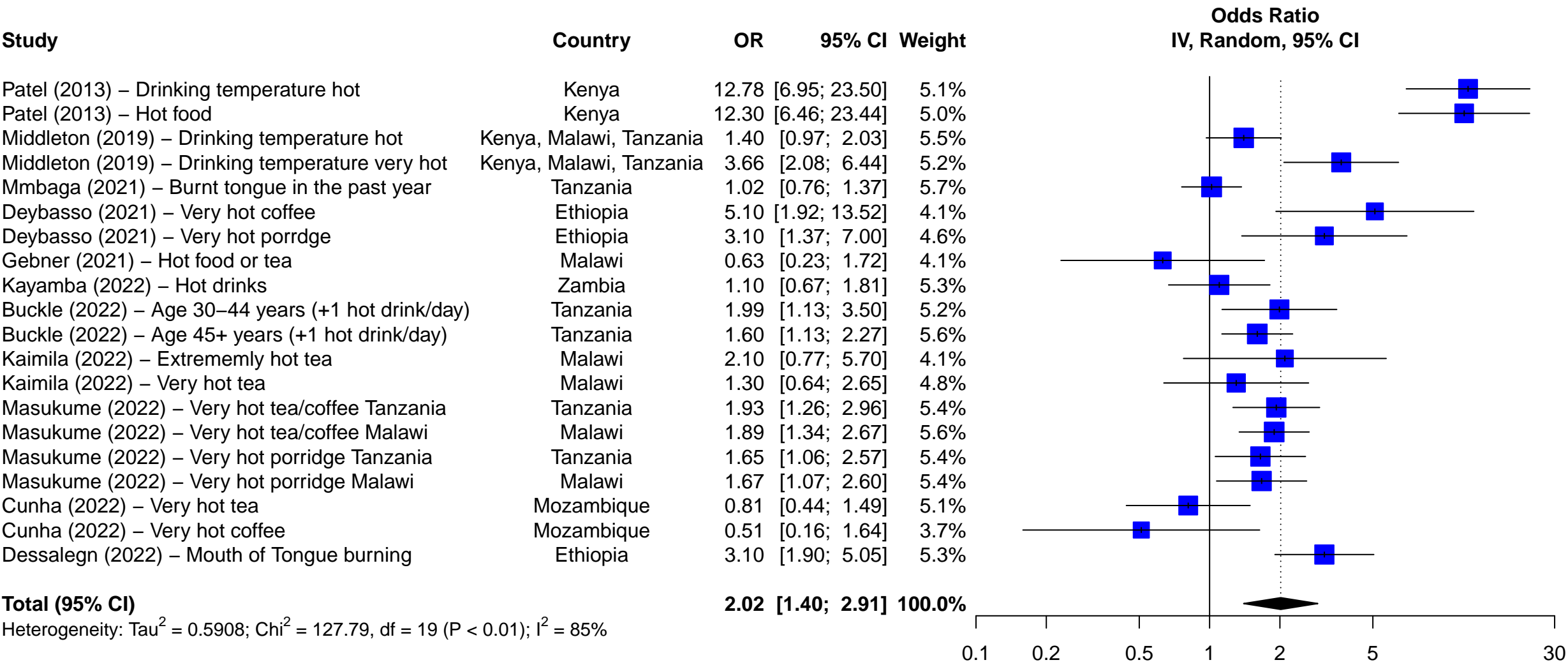

Supplement: Supplementary file 5 — Additional file 5. Effect of combined tobacco and alcohol use on ESCC in Africa. This diagram is a forest plot showing pooled analysis of all studies (no exclusion of outliers). Study ID gives the first author and the year of the publication. OR, odds ratio; CI, confidence interval. PDF. [file 12889_2023_16629_MOESM5_ESM.pdf]

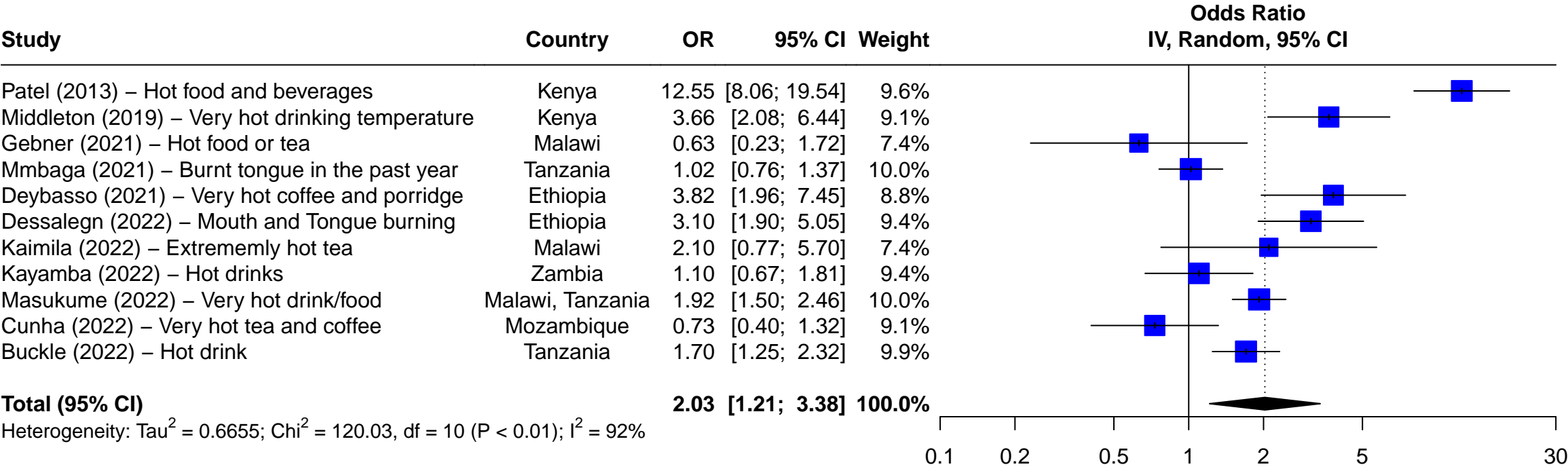

Supplement: Supplementary file 6 — Additional file 6. Effect of hot food and beverage on ESCC in Africa. This diagram is a forest plot showing pooled analysis of all studies (no exclusion of outliers). Study ID gives the first author and the year of the publication. OR, odds ratio; CI, confidence interval. PDF. [file 12889_2023_16629_MOESM6_ESM.pdf]

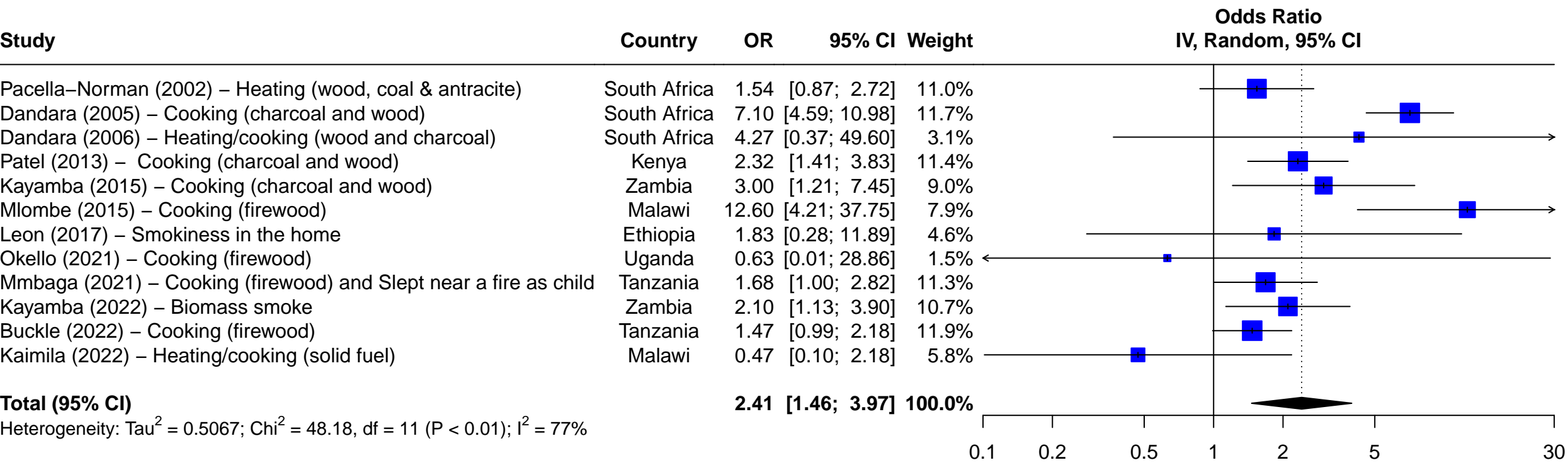

Supplement: Supplementary file 7 — Additional file 7. Effect of PAH on ESCC in Africa. This diagram is a forest plot showing pooled analysis of all studies (no exclusion of outliers). Study ID gives the first author and the year of the publication. OR, odds ratio; CI, confidence interval; PAH, polycyclic aromatic hydrocarbons. PDF. [file 12889_2023_16629_MOESM7_ESM.pdf]

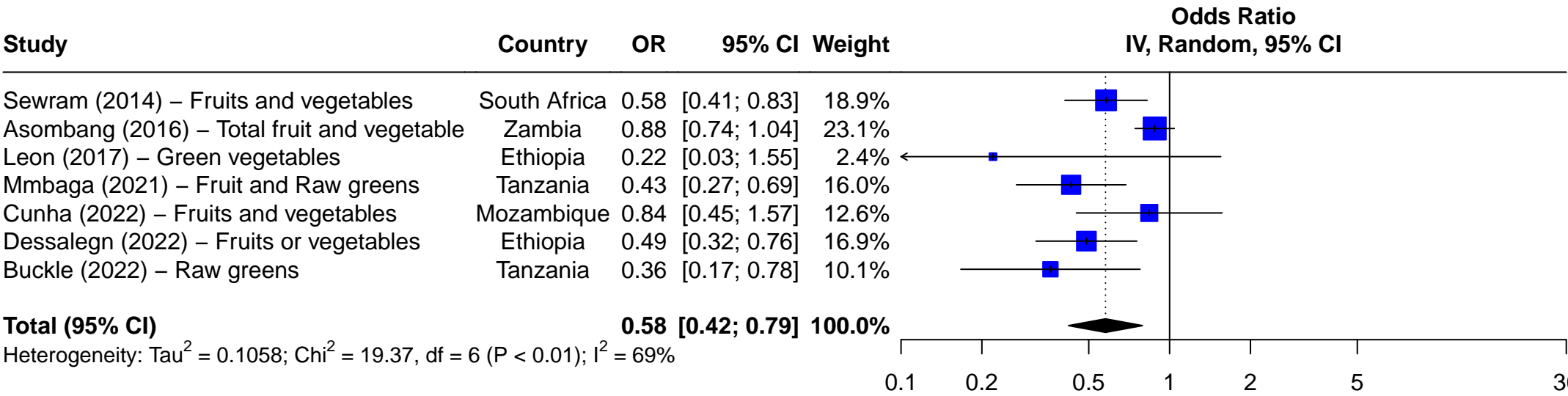

Supplement: Supplementary file 8 — Additional file 8. Effect of fruit and vegetable consumption on ESCC in Africa. This diagram is a forest plot showing pooled analysis of all studies (no exclusion of outliers). Study ID gives the first author and the year of the publication. OR, odds ratio; CI, confidence interval. PDF. [file 12889_2023_16629_MOESM8_ESM.pdf]

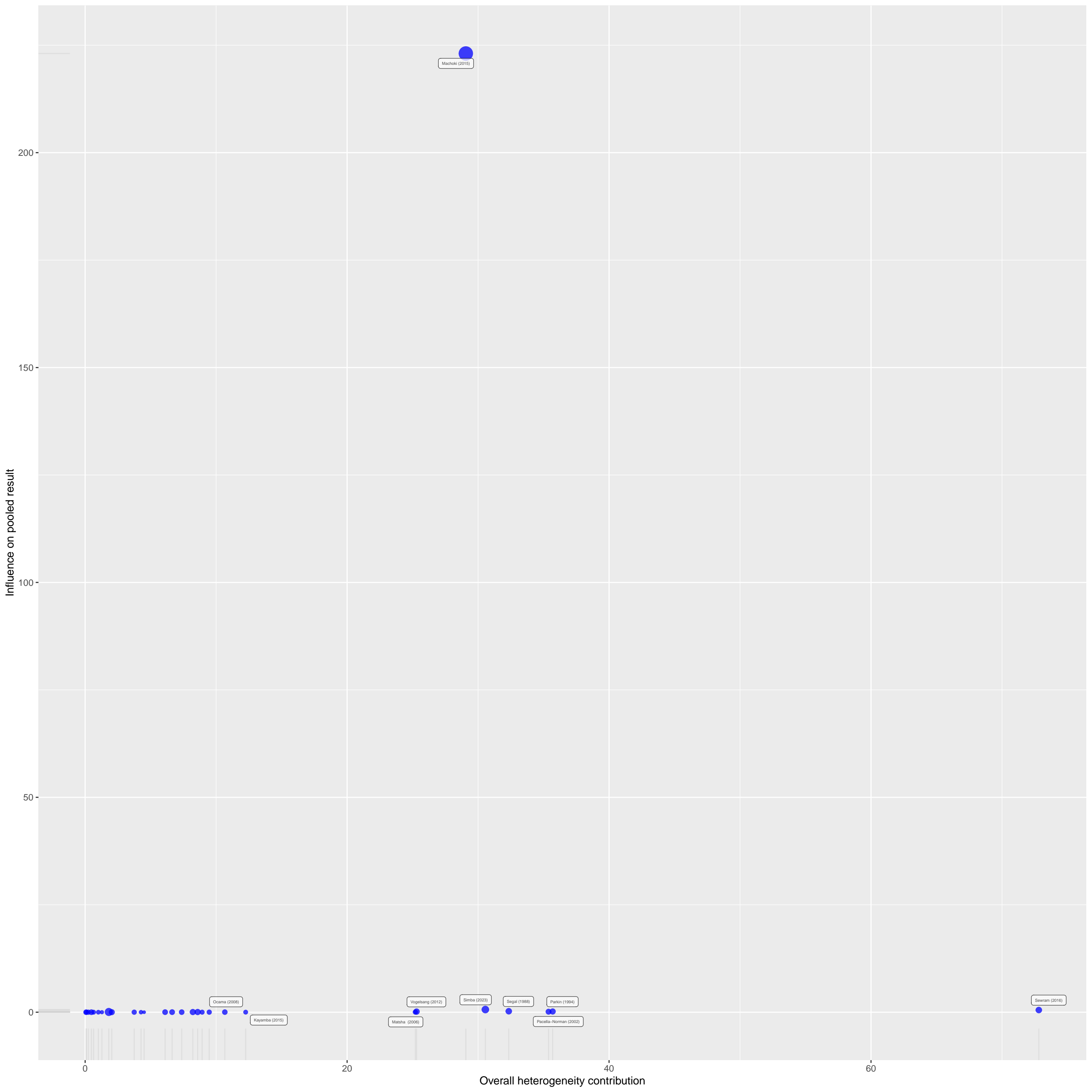

Supplement: Supplementary file 9 — Additional file 9. Baujat plot for tobacco use from outlier and influence analysis. PDF. [file 12889_2023_16629_MOESM9_ESM.pdf]

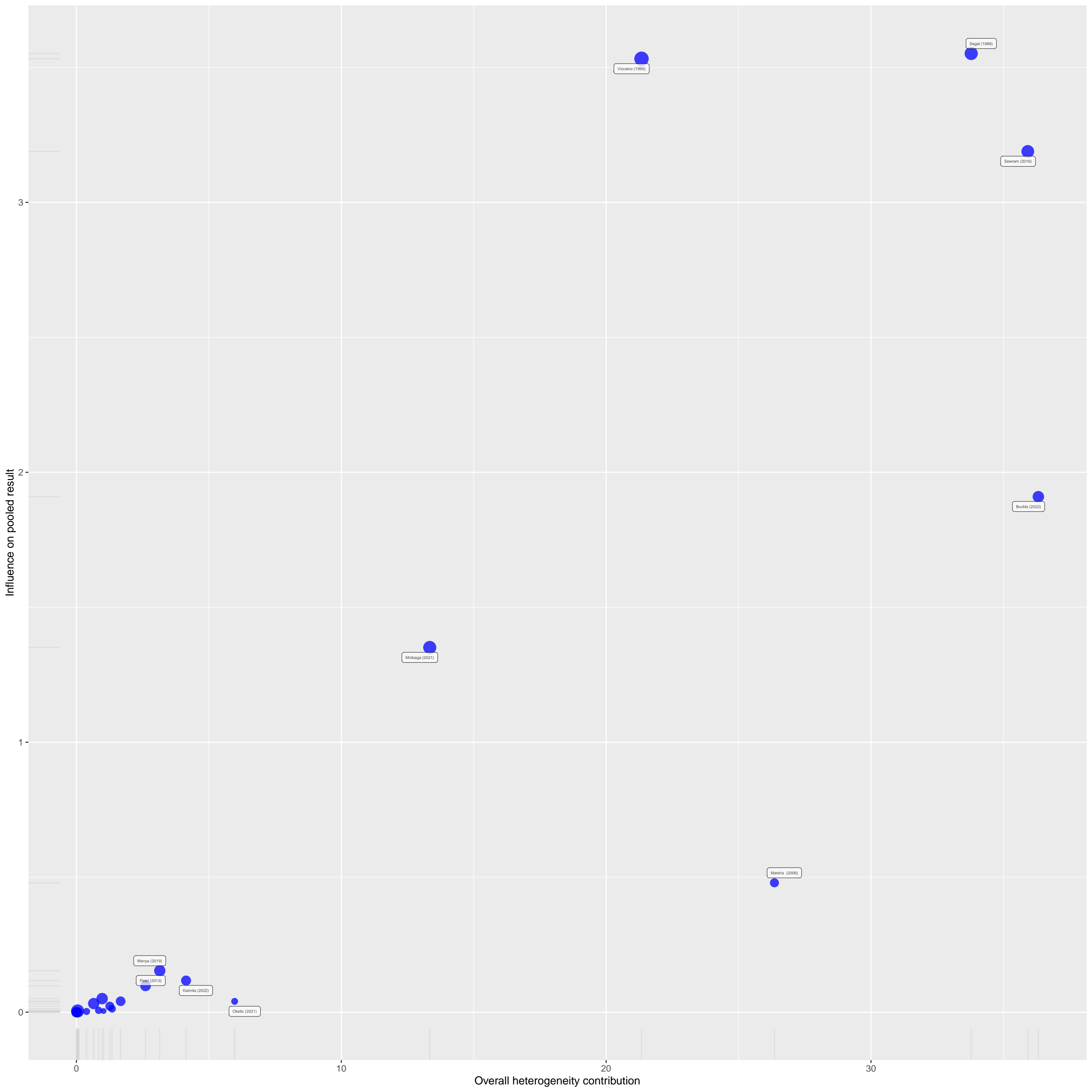

Supplement: Supplementary file 10 — Additional file 10. Baujat plot for alcohol use from outlier and influence analysis. PDF. [file 12889_2023_16629_MOESM10_ESM.pdf]

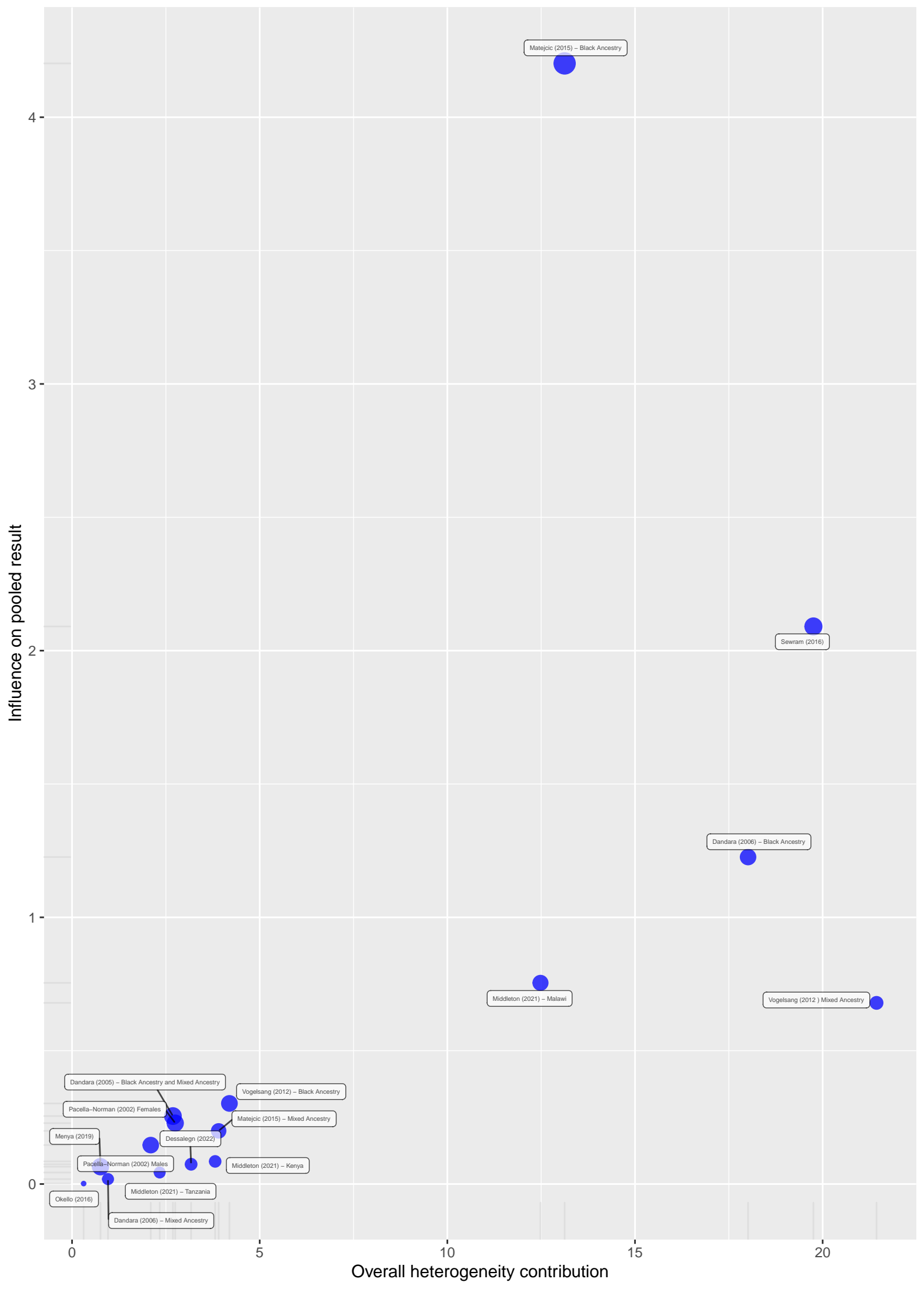

Supplement: Supplementary file 11 — Additional file 11. Baujat plot for combined tobacco and alcohol use from outlier and influence analysis. PDF. [file 12889_2023_16629_MOESM11_ESM.pdf]

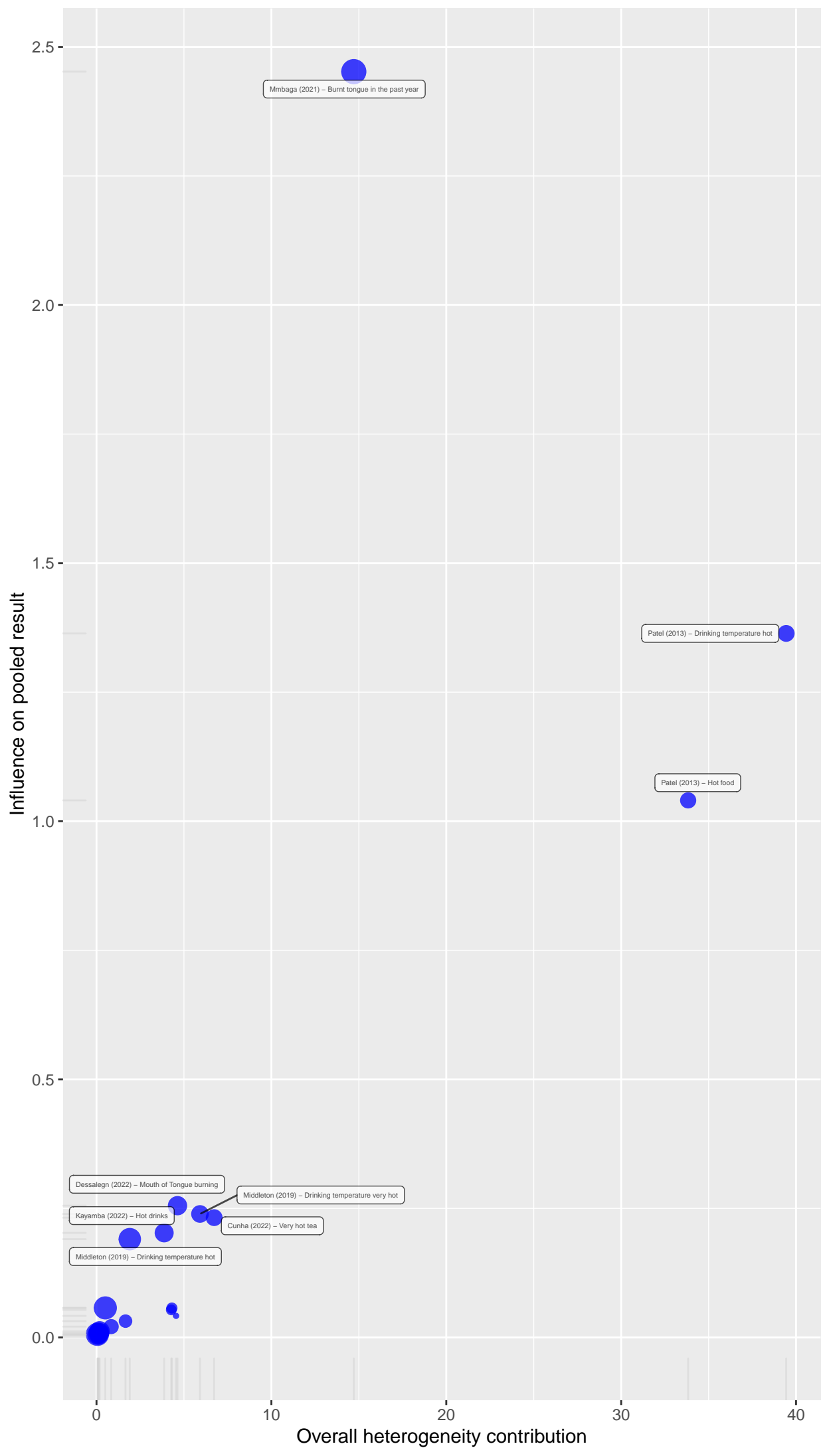

Supplement: Supplementary file 12 — Additional file 12. Baujat plot for hot food and beverage consumption from outlier and influence analysis. PDF. [file 12889_2023_16629_MOESM12_ESM.pdf]

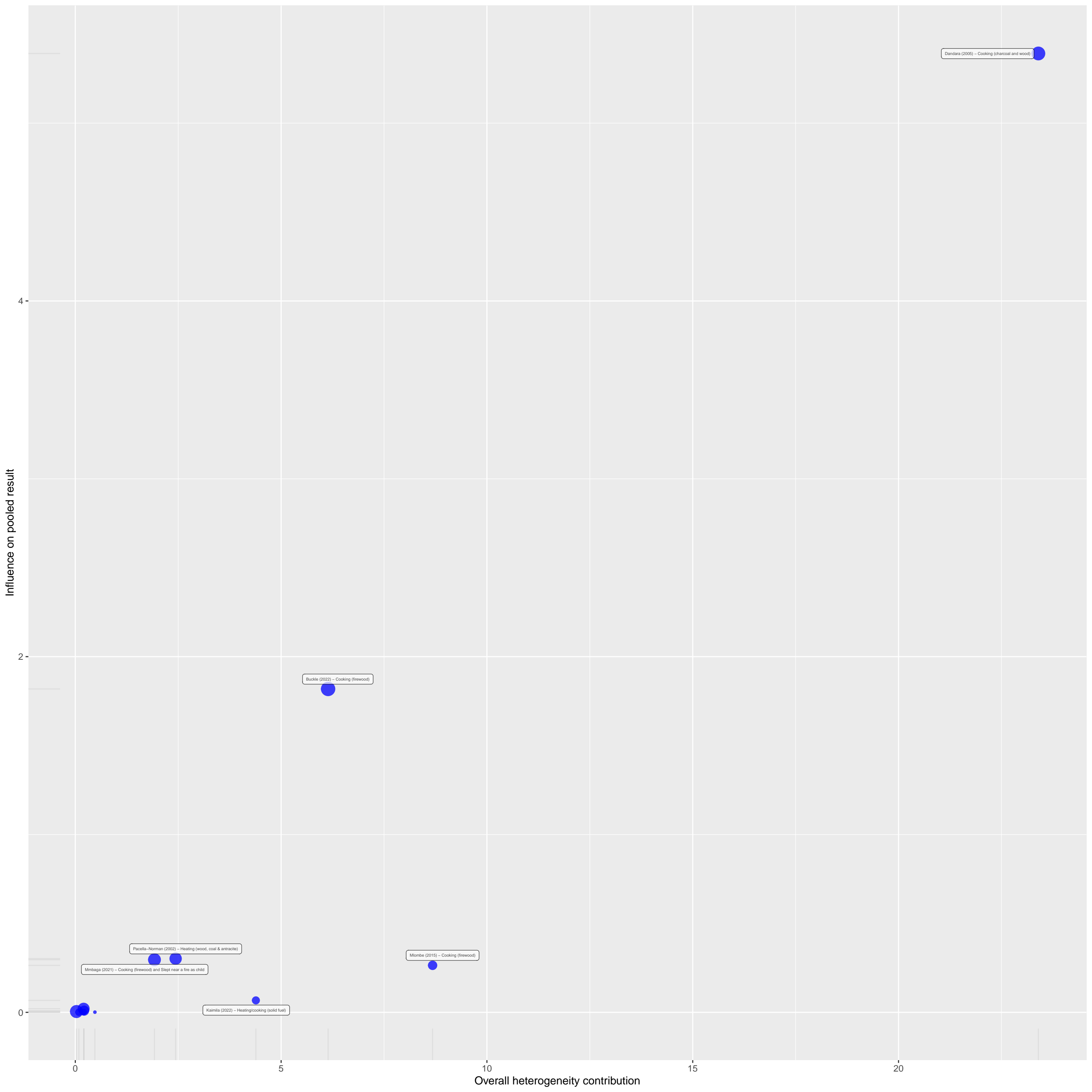

Supplement: Supplementary file 13 — Additional file 13. Baujat plot for PAH exposure from outlier and influence analysis. PAH, polycyclic aromatic hydrocarbons. PDF. [file 12889_2023_16629_MOESM13_ESM.pdf]

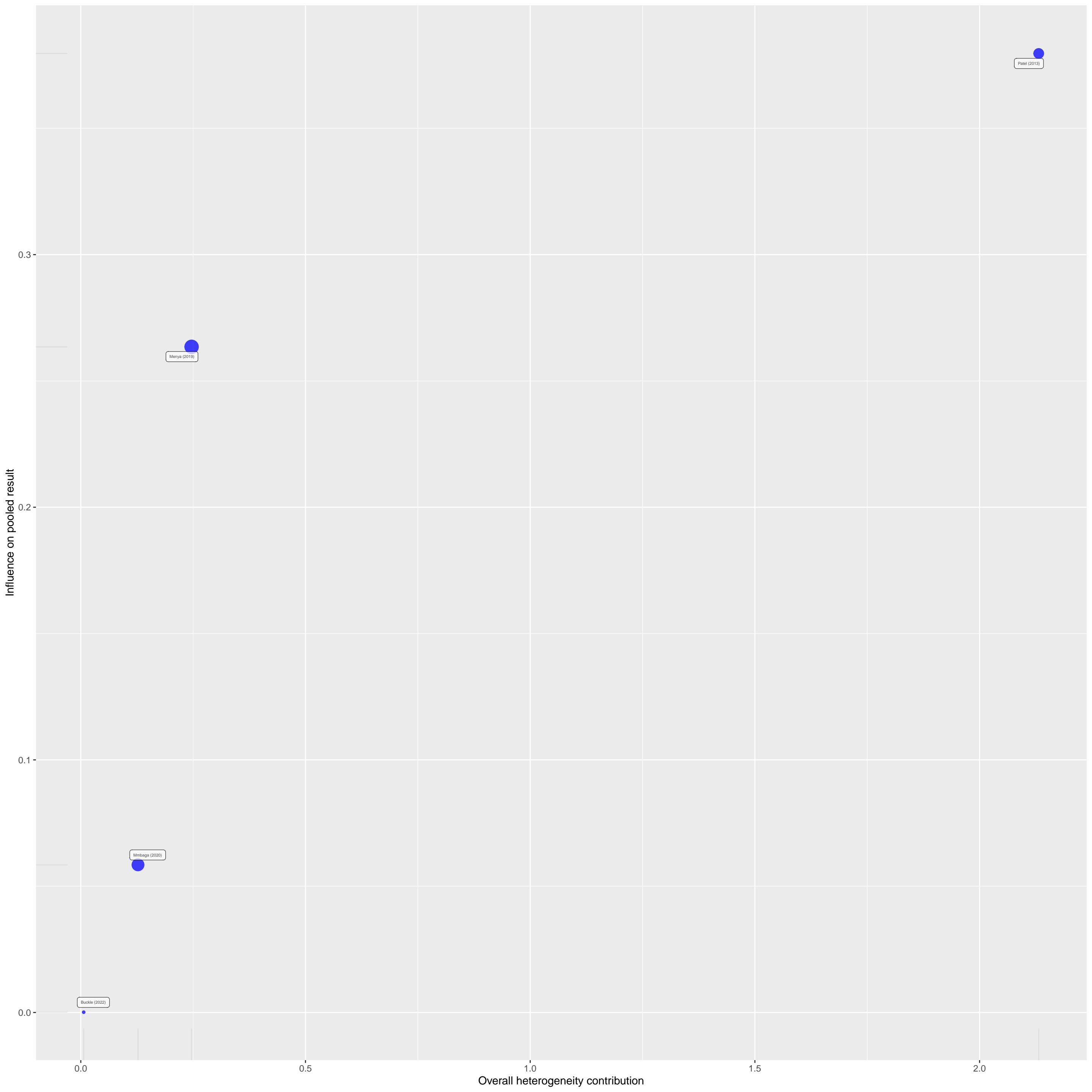

Supplement: Supplementary file 14 — Additional file 14. Baujat plot for oral health from outlier and influence analysis. PDF. [file 12889_2023_16629_MOESM14_ESM.pdf]

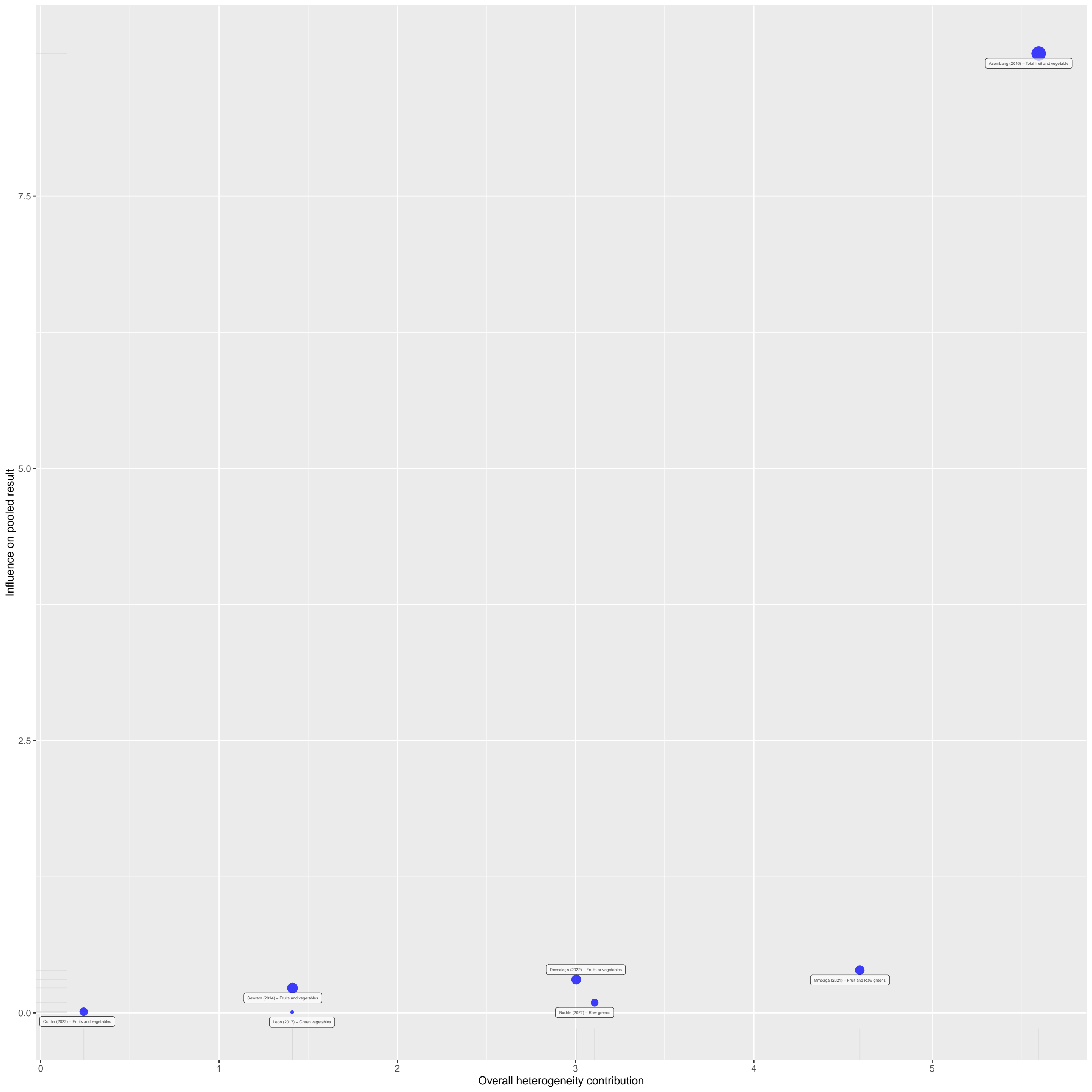

Supplement: Supplementary file 15 — Additional file 15. Baujat plot for fruit and vegetable consumption from outlier and influence analysis. PDF. [file 12889_2023_16629_MOESM15_ESM.pdf]

Sorted by Effect Size

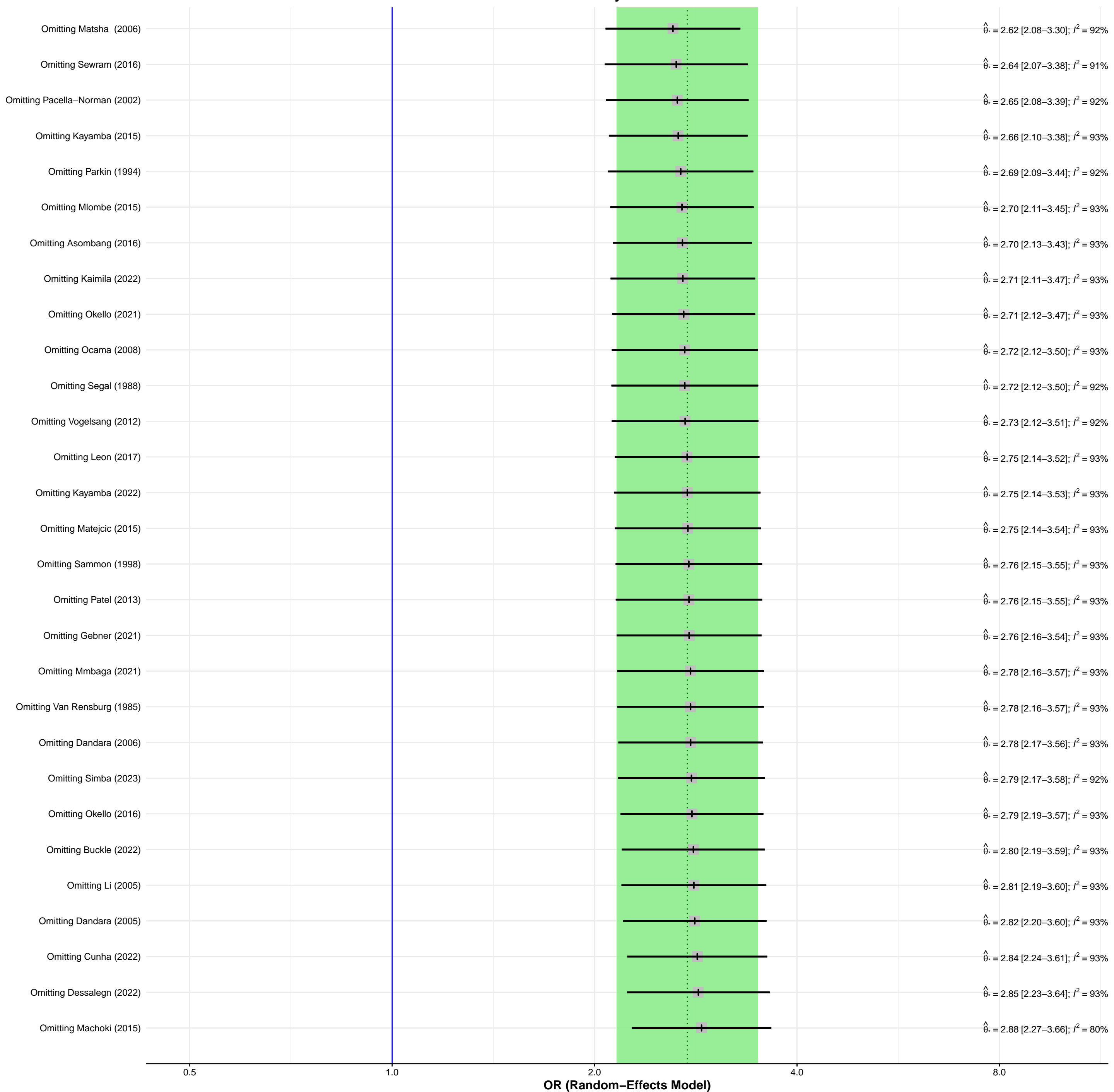

Supplement: Supplementary file 16 — Additional file 16. Sensitivity plot for tobacco use. Sensitivity analysis was done using the "Leave-One-Out" influence analysis on studies included in the final meta-analysis. This was done to determine which study may have had an excessive influence on the overall effect size. PDF. [file 12889_2023_16629_MOESM16_ESM.pdf]

Sorted by Effect Size

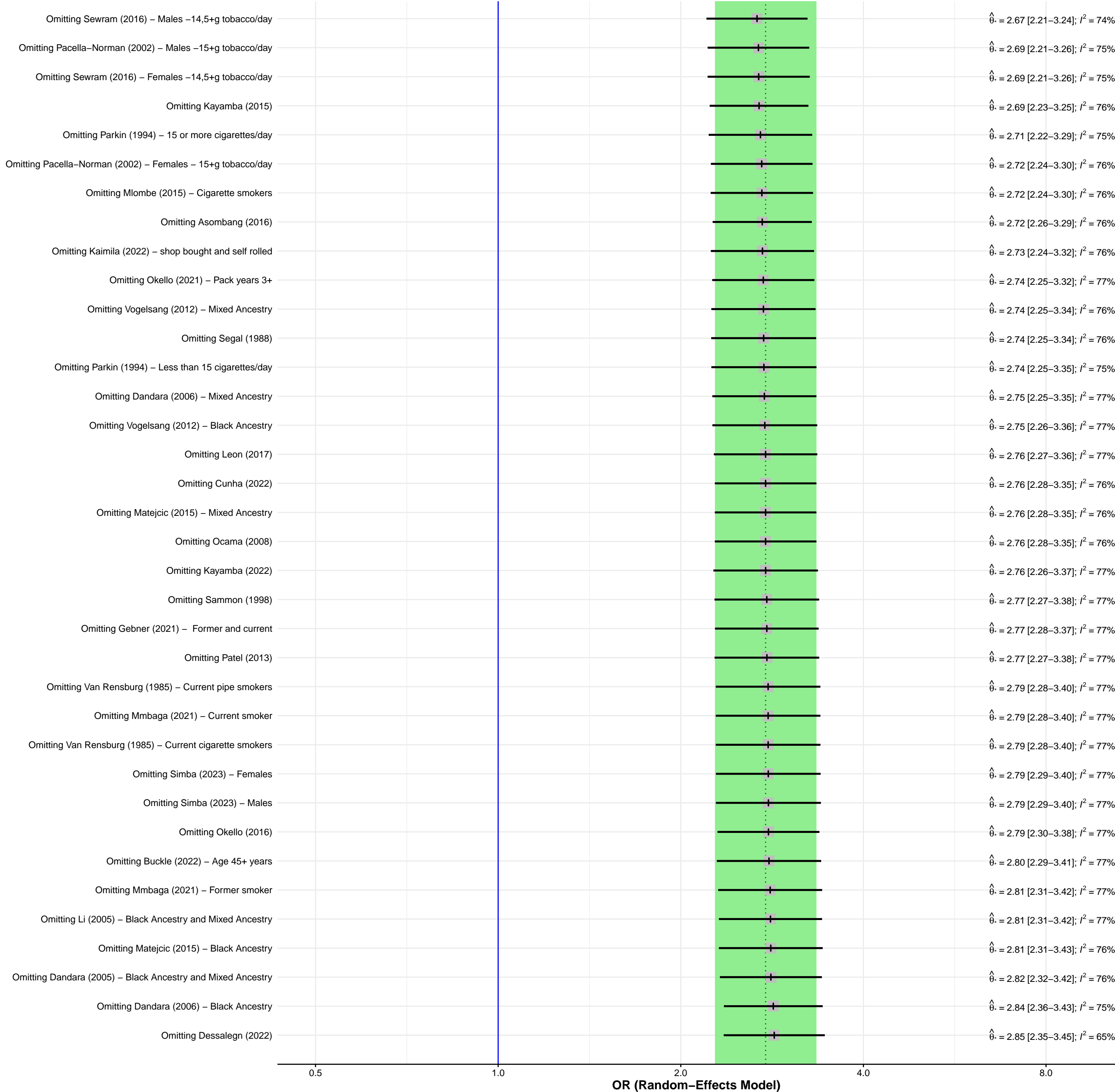

Supplement: Supplementary file 17 — Additional file 17. Sensitivity plot for alcohol use. PDF. [file 12889_2023_16629_MOESM17_ESM.pdf]

Sorted by Effect Size

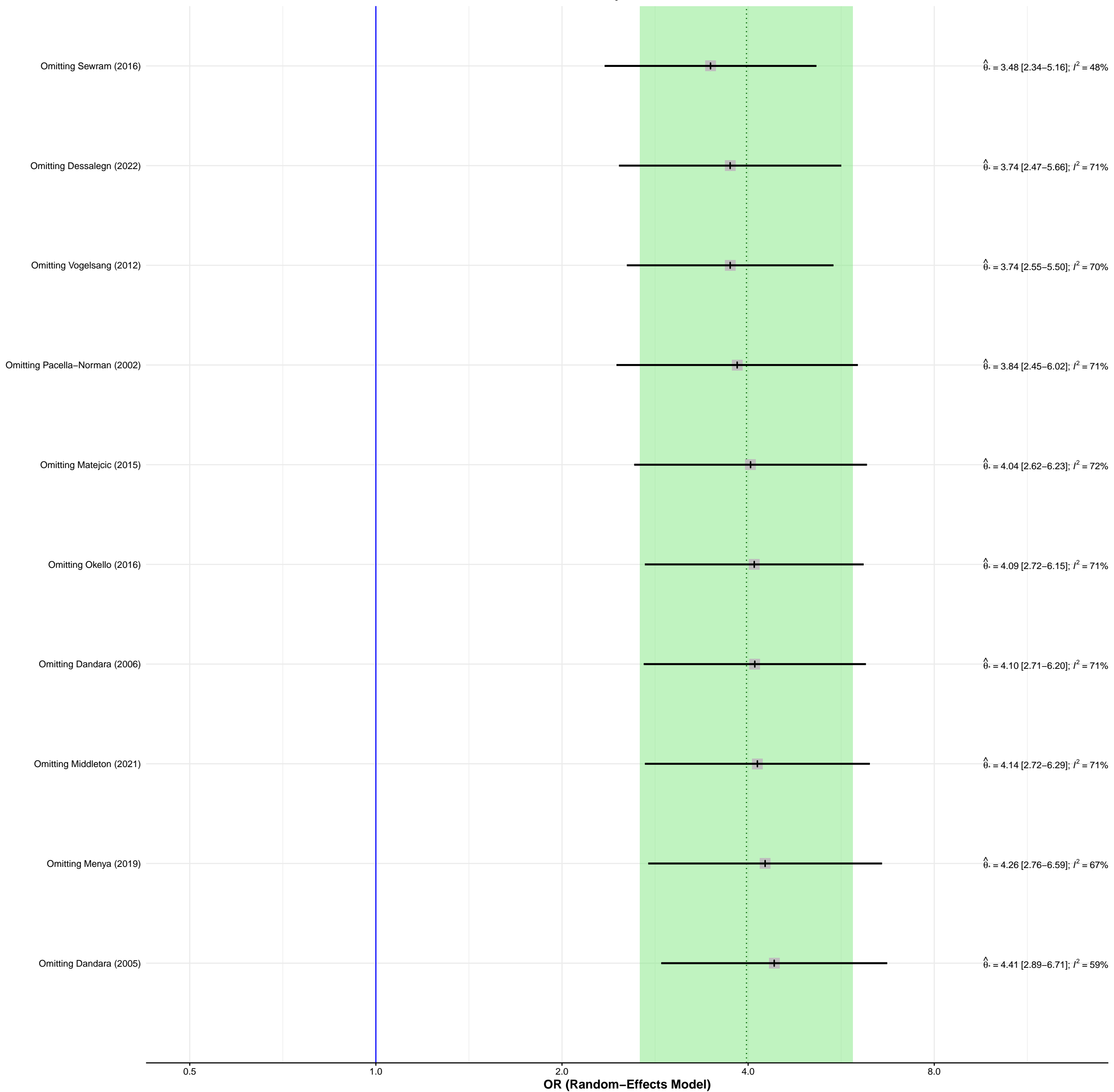

Supplement: Supplementary file 18 — Additional file 18. Sensitivity plot for combined alcohol and tobacco use. PDF. [file 12889_2023_16629_MOESM18_ESM.pdf]

Sorted by Effect Size

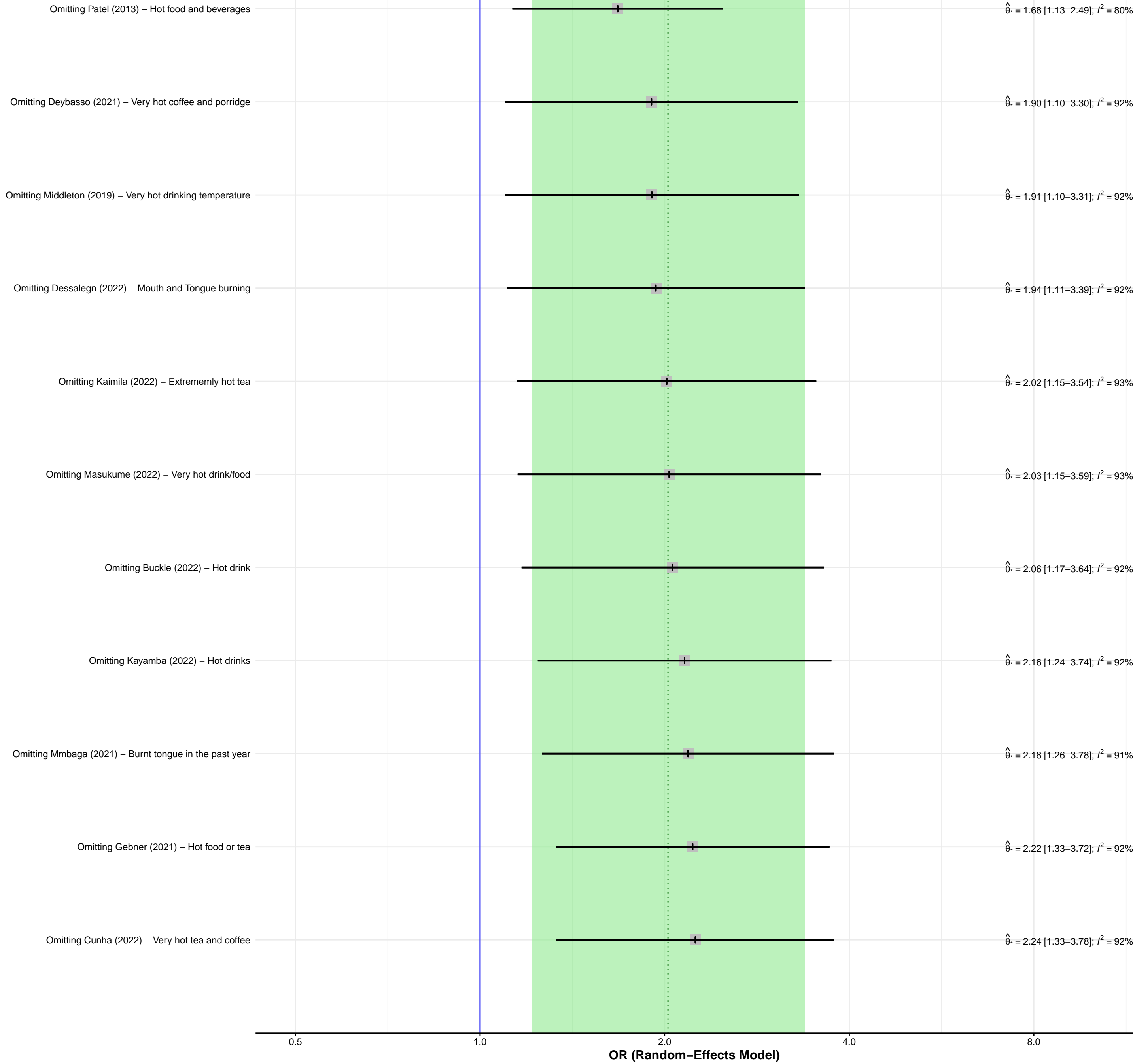

Supplement: Supplementary file 19 — Additional file 19. Sensitivity plot for hot food and beverage consumption. PDF. [file 12889_2023_16629_MOESM19_ESM.pdf]

Sorted by Effect Size

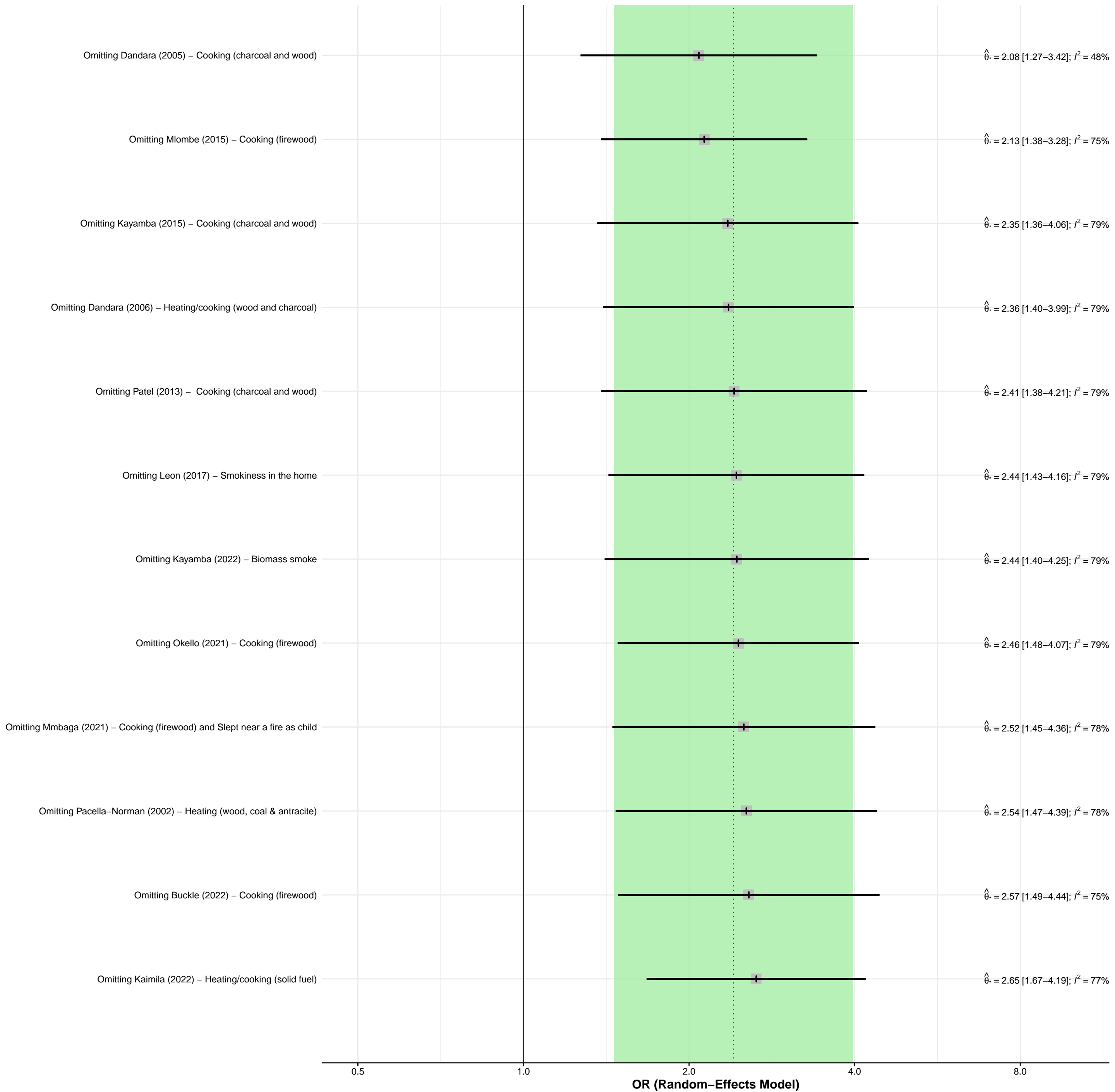

Supplement: Supplementary file 20 — Additional file 20. Sensitivity plot for combined PAH exposure. PDF. [file 12889_2023_16629_MOESM20_ESM.pdf]

Sorted by Effect Size

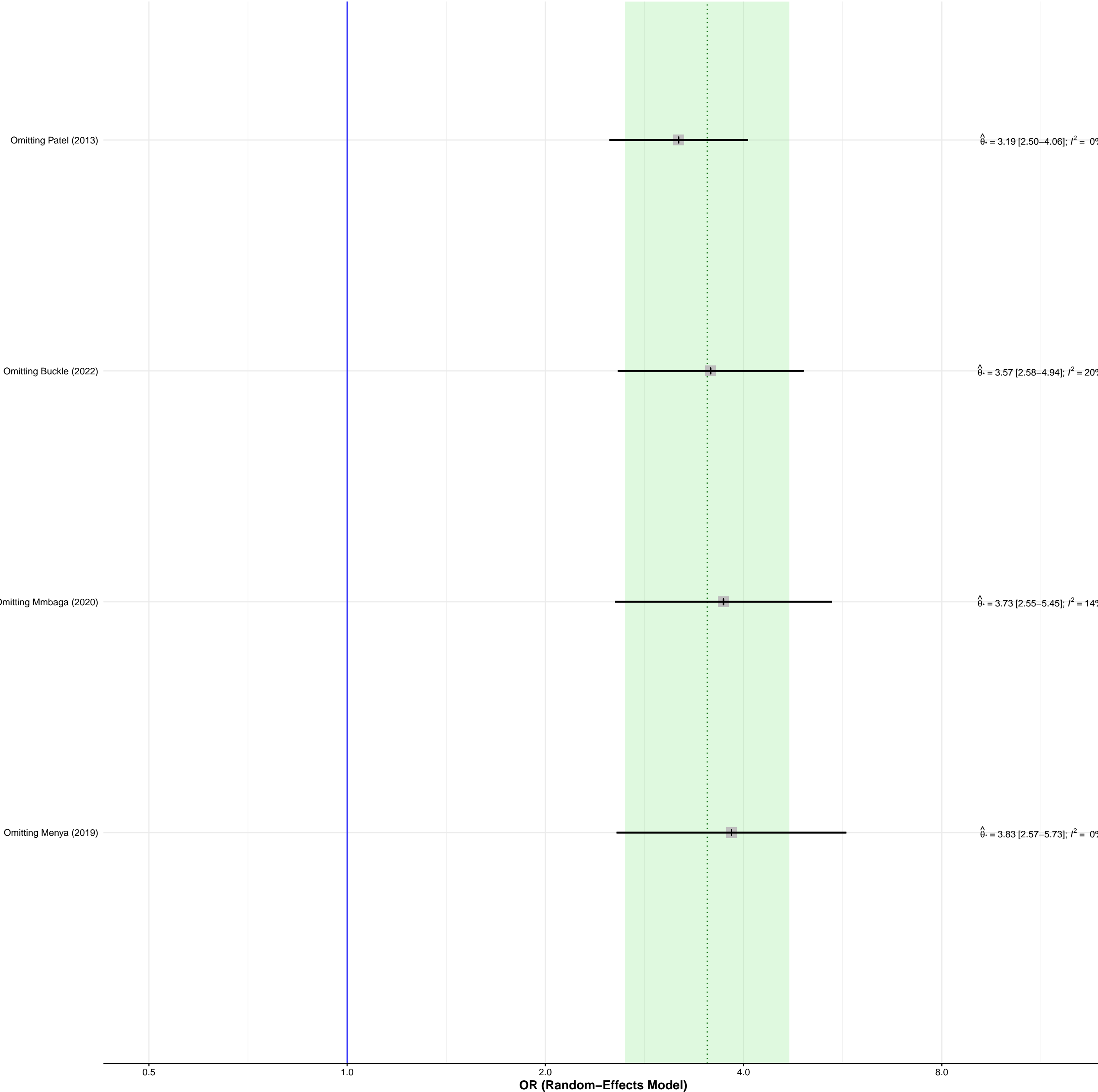

Supplement: Supplementary file 21 — Additional file 21. Sensitivity plot for oral health. PDF. [file 12889_2023_16629_MOESM21_ESM.pdf]

Sorted by Effect Size

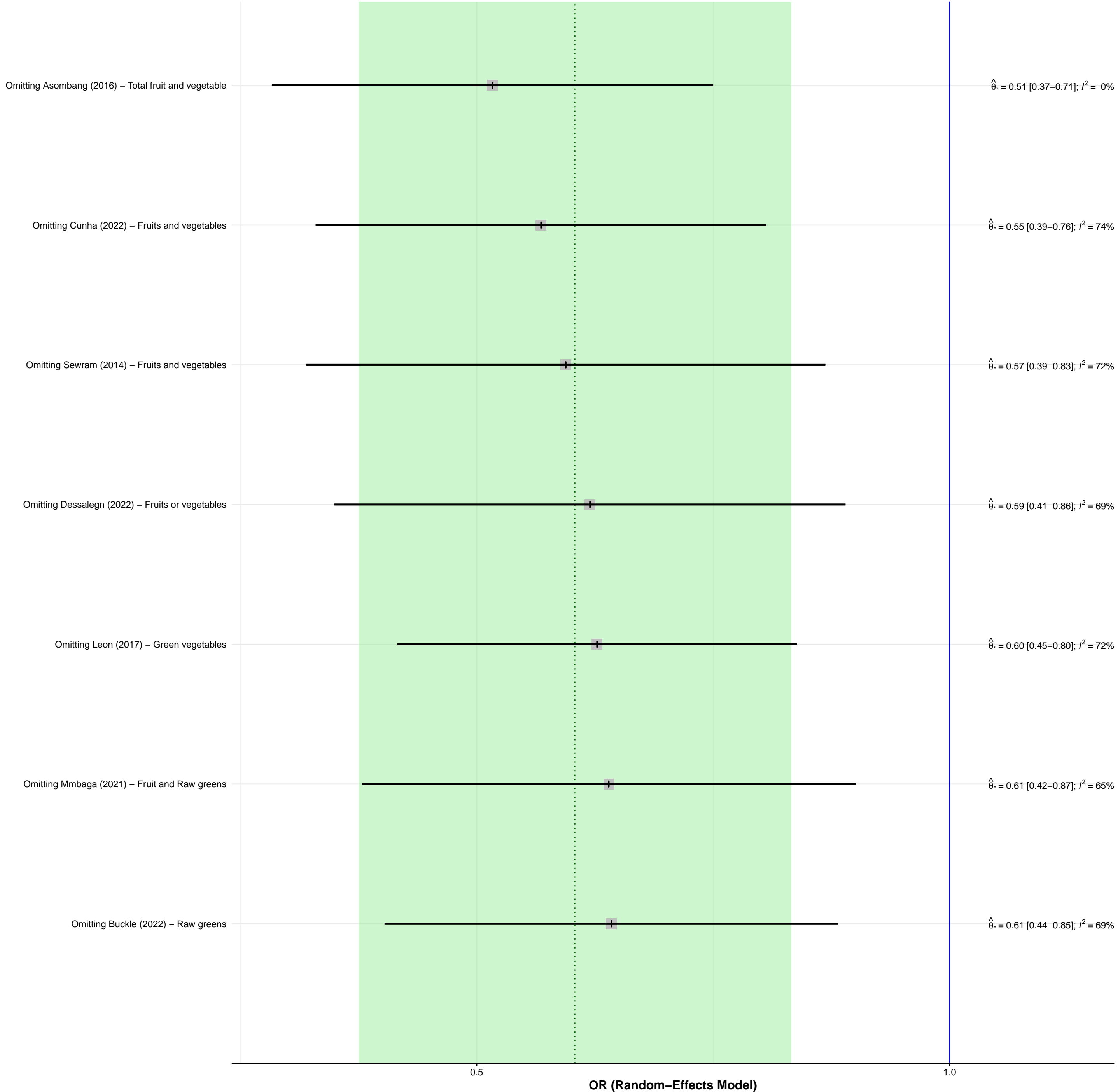

Supplement: Supplementary file 22 — Additional file 22. Sensitivity plot for fruit and vegetable consumption. PDF. [file 12889_2023_16629_MOESM22_ESM.pdf]
